# Comparative Effectiveness of Antivirals and Monoclonal Antibodies for Treating COVID‐19 Patients Infected With Omicron Variant: A Systematic Review and Network Meta‐Analysis

**DOI:** 10.1111/irv.70065

**Published:** 2024-12-25

**Authors:** Kristy T. K. Lau, Xi Xiong, Carlos K. H. Wong, Ivan C. H. Au, Angel Y. C. Lui, Gavin Y. T. Tsai, Tingting Wu, Lanlan Li, Eric H. Y. Lau, Benjamin J. Cowling, Gabriel M. Leung

**Affiliations:** ^1^ Department of Family Medicine and Primary Care, School of Clinical Medicine, Li Ka Shing Faculty of Medicine University of Hong Kong Hong Kong SAR China; ^2^ Department of Pharmacology and Pharmacy, Li Ka Shing Faculty of Medicine The University of Hong Kong Hong Kong SAR China; ^3^ Research Department of Practice and Policy, School of Pharmacy University College London London UK; ^4^ Laboratory of Data Discovery for Health Limited (D^2^4H), Hong Kong Science Park, new Territories Hong Kong SAR China; ^5^ Department of Infectious Disease Epidemiology and Dynamics London School of Hygiene and Tropical Medicine London UK; ^6^ The Hong Kong Jockey Club Global Health Institute Hong Kong SAR China; ^7^ School of Public Health, Li Ka Shing Faculty of Medicine University of Hong Kong Hong Kong SAR China; ^8^ Department of Medicine, Li Ka Shing Faculty of Medicine University of Hong Kong Hong Kong SAR China; ^9^ WHO Collaborating Centre for Infectious Disease Epidemiology and Control, School of Public Health, Li Ka Shing Faculty of Medicine University of Hong Kong Hong Kong SAR China; ^10^ Institute for Health Transformation, Faculty of Health Deakin University Melbourne Australia

**Keywords:** antiviral, COVID‐19, monoclonal antibody, Omicron, SARS‐CoV‐2, treatment

## Abstract

Antiviral drugs likely remain effective against the SARS‐CoV‐2 Omicron variant, while monoclonal antibody (mAb) therapies have experienced drops in neutralizing ability. This systematic review and network meta‐analysis aims to estimate the comparative effectiveness of antivirals and mAb therapies for treating COVID‐19 patients infected with Omicron, capturing primarily acute outcomes. We searched multiple databases from July 4 to July 19, 2022, with updates through November 4, 2022. Studies comparing the effectiveness of antivirals or mAb to either nonuser controls or other treatments were included. Risk of bias was assessed using the Cochrane RoB 2 and ROBINS‐I tools. Data extraction and verification involved five independent researchers. Among 39 studies (727,893 individuals with COVID‐19, including 38 nonrandomized trials), nirmatrelvir/ritonavir and sotrovimab were associated with lower risks of mortality (HR = 0.317, 95% credible intervals [CrI] = 0.144–0.678; HR = 0.176, 95%CrI = 0.052–0.527) and hospitalization (HR = 0.479, 95%CrI = 0.319–0.711; HR = 0.489, 95%CrI = 0.293–0.797) compared with nonuser controls. Remdesivir users were associated with a lower risk of hospitalization (HR = 0.367, 95%CrI = 0.147–0.868) but not mortality. Molnupiravir and bebtelovimab showed no significant benefits for these outcomes. In conclusion, among individuals infected with COVID‐19 during the Omicron wave, mortality risk was lower with nirmatrelvir/ritonavir or sotrovimab use, whereas hospitalization was reduced with nirmatrelvir/ritonavir, remdesivir, or sotrovimab. Sotrovimab and nirmatrelvir/ritonavir were effective against Omicron B.1.1.529/BA.1 and BA.2/BA.4/BA.5 subvariants, respectively. A key limitation is that findings rely on data from the last search and may be impacted by potential changes in mortality risk due to immune evasion by emerging variants, highlighting the need for ongoing randomized trials across variants and populations.

**Trial Registration:**

The study was registered on PROSPERO, CRD42022351508.

## 1 Introduction

The Omicron variant of COVID‐19 (B.1.1.529) was first reported to the World Health Organization (WHO) on November 24, 2021 [1]. The variant, first identified in Botswana and South Africa, quickly superseded the Delta variant as the dominant circulating SARS‐CoV‐2 variant in the world due to its very high transmission rate, as well as immune evasion from vaccines and neutralizing antibodies [2]. During the Omicron wave in the United States, Omicron variants accounted for 95% of all sequenced COVID‐19 cases [3]. The variant is further divided into subvariants as it evolves, including BA.1, BA.2, BA.4, and BA.5 [4]. Early in January 2022, BA.1 reached its peak before being superseded by BA.2 [5]. Following that, BA.2 represented 86% of total COVID‐19 cases reported to the WHO, becoming the more dominant subvariant during February–March 2022 [6]. In late 2022, BA.5, BQ.1, and BQ.1.1 have emerged and accounted for >80% of new COVID‐19 cases in the US [7].

Following the identification of the Omicron variant and its subvariants, in vitro studies have been conducted to determine the neutralizing abilities of pharmaceutical interventions against this variant of concern (VOC). Results have suggested that antiviral drugs, that is, remdesivir, molnupiravir, and nirmatrelvir/ritonavir, likely remain effective against most notable variants in circulation [4]. However, monoclonal antibody (mAb) therapies have experienced drops in their neutralizing ability against Omicron [8, 9]. Casirivimab/imdevimab may be ineffective against BA.1, alongside conflicting evidence regarding its in vitro efficacy against BA.2 and subsequent subvariants [10, 11, 12]. Although sotrovimab has demonstrated neutralizing ability against BA.1, it is generally ineffective against BA.2 and subsequent subvariants [4, 13]. Bebtelovimab potently neutralizes Omicron B.1.1.529, BA.2, BA.2.12.1, BA.2.75, BA.4, and BA.5 but not BQ.1 and BQ.1.1 subvariants [4, 14, 15, 16, 17]. Additionally, Omicron subvariants, except for BA.2.75, are found to be less susceptible to tixagevimab/cilgavimab [4, 10, 14, 15]. It is important to note that direct virus neutralization is only one of several antiviral mechanisms expected for pharmaceutical interventions against VOC to act in vivo; therefore, changes in in vitro neutralization potency against different variants may not fully represent the actual antiviral effectiveness observed in clinical settings [18]. For example, in vivo studies have demonstrated the effectiveness of sotrovimab in clinical settings when Omicron BA.2 was the predominant variant, despite previous in vitro studies suggesting it is generally ineffective against BA.2 [4, 13, 19].

Although there are several systematic reviews and meta‐analyses published for the evaluation of pharmaceutical interventions against COVID‐19 [20, 21, 22, 23], they did not differentiate treatment groups by VOC and involved mostly trials before the Omicron era. At the time of writing, there is a lack of head‐to‐head comparison of the efficacy and effectiveness of pharmacological treatments against clinical outcomes concerning the Omicron variant. Therefore, this systematic review and network meta‐analysis aims to summarize the direct and indirect evidence of the clinical effectiveness of different antivirals and mAb therapies in treating COVID‐19 patients infected with the Omicron variant and its sublineages.

## 2 Methods

### 2.1 Data Sources and Searches

We used PubMed, Embase, Cochrane COVID‐19 study register, VIEW‐hub study repository, and WHO COVID‐19 database to capture a comprehensive range of published and pre‐print studies, encompassing clinical trials and observational studies with a focus on COVID‐19‐related research and real‐world data. Studies were also identified from citations of included studies. Identification of studies occurred from July 4 to July 19, 2022. Additional hand search and study screening were completed by November 4, 2022.

The protocol of this study is registered with PROSPERO, number CRD42022351508. Results are reported in accordance with the PRISMA extension statement for network meta‐analysis [24].

### 2.2 Study Selection

In this review, we included original, randomized trials and observational studies (i.e., case–control studies and cohort studies) of human participants investigating the effectiveness of antivirals and mAb therapies against clinical outcomes during the Omicron predominant period (defined as November 24, 2021, and later), in preprint or publication. We excluded systematic reviews, meta‐analyses, preclinical studies, commentaries, editorials, correspondence without original findings, case series or case reports, and observational studies without a comparison or control group; studies investigating vaccine immunogenicity, safety, and efficacy; studies investigating the effectiveness of drug treatments other than antivirals and mAb therapies; studies investigating pharmaceutical pre‐ and post‐exposure prophylaxis of COVID‐19; studies investigating post‐COVID‐19 conditions; and non‐English studies. To address potential duplication from multiple databases, we used EndNote to systematically remove duplicates before screening. The identified studies were independently screened by four researchers (KTKL, XX, GYTT, and AYCL) by title, then by abstract, and eventually by full text. Any discrepancies during study selection were resolved through discussion with the senior author (C.K.H.W.) to ensure consistency and thorough evaluation. Search strategies developed for this study are outlined in Appendix S1.

### 2.3 Data Extraction and Quality Assessment

Regarding the use of antivirals or mAb, first author's last name and country, study design with variables controlled, Omicron subvariants, observation period, study population (age and special patient population), inclusion and exclusion criteria, sample size, patient setting, disease severity, dosage, time from symptom onset to treatment initiation, COVID‐19 vaccination status, disease outcomes, and effect measures in terms of odds ratios (ORs)/hazard ratios (HRs)/incidence rate ratios (IRRs) (coupled with 95% confidence intervals and *p*‐values). OR/HR/IRR (coupled with 95% CI and *p*‐values) were extracted by five independent researchers (K.T.K.L., I.C.H.A., G.Y.T.T., A.Y.C.L., and X.X.). Any discrepancies were reconciled and resolved by discussion with the senior author of the study (C.K.H.W.).

Bias assessment and quality assessment for randomized controlled trials (RCTs) and observational studies were carried out using the Cochrane Risk of Bias 2 (RoB 2) tool [25] and ROBINS‐I tool (for nonrandomized studies of interventions) [26], respectively. The RoB 2 tool (Figure S1) assesses the risk of bias for randomized trials in five domains, namely, bias arising from the randomization process, bias due to deviations from intended interventions, bias due to missing outcome data, bias in the measurement of the outcome, and bias in the selection of the reported result [25]. The ROBINS‐I tool (Figure S1) assesses the risk of bias due to confounding, bias in the selection of participants into the study, bias in the classification of interventions, bias due to deviations from intended intervention, bias due to missing data, bias in the measurement of outcomes, and bias in the selection of the reported results [26]. Risk of bias assessments were independently conducted by four researchers (T.T.W., L.L.L., G.Y.T.T., and A.Y.C.L.), with any discrepancies resolved through discussion with the senior author (C.K.H.W.) to ensure a robust quality assessment process.

### 2.4 Data Synthesis and Analysis

The outcomes of this study were (1) mortality from any causes and (2) hospitalization from all causes (COVID‐19 or non‐COVID‐19 related).

We performed a random‐effect network meta‐analysis using all available direct and indirect comparisons to estimate the pooled HRs with 95% credible intervals (CrIs) for all pairwise treatment comparisons, where CrIs crossing 1 indicate no significant difference between treatments. The use of 95% CrIs, rather than traditional confidence intervals, reflects the Bayesian approach applied in network meta‐analysis, providing a range within which the true effect size is likely to fall with 95% probability. Control refers to the “nonuser group” in new user design studies, whereas in active comparator design studies, all active comparators were included in analyses. Arm‐level and relative effect data were extracted from eligible studies for prespecified outcomes in each treatment group. Arm‐level data refer to the sample size and the total number of events, and relative effect data refer to the HR in our analyses. In order to avoid double counting, only relative effect data were included in the analysis for an individual study that reports both arm‐level and relative effect data (Table S1) [27].

We established a consistency model assuming no discrepancy between direct and indirect evidence for comparisons of each treatment in random effect with package “gemtc” [28]. There is a potential for bias if the time points used in the systematic review are subjectively chosen or selectively reported by the trialist, particularly if they coincide with periods of maximum or minimum difference between intervention groups [29]. To account for the dichotomous outcome and different follow‐up times between studies, we conducted an analysis by fitting a model with a complementary log–log link function and binomial likelihood function. Heterogeneity was accounted for by applying empirical priors based on Turner et al. [30], providing a robust foundation for modeling between‐study variance, supplemented by subgroup analyses to identify potential sources of variability. We employed uninformative prior distributions as automatically selected by *gemtc* to perform an objective, data‐driven analysis given that we did not have a robust understanding of the treatment effect of COVID‐19 therapies. All models were run in four Markov chain Monte Carlo chains, and the convergence was checked by using the Gelman–Rubin statistic [31] after 50,000 iterations and 20,000 samples burn‐in phase. We used the node‐splitting approach [32] to assess statistical evidence for inconsistency, defined as a discrepancy between direct and indirect evidence (Figure S2). We applied Egger's test to examine publication bias.

We performed sensitivity analyses to evaluate the robustness of results according to sample size (excluding small studies with sample size less than 100) and risk of bias (excluding studies with critical/high risk of bias in one or more domains or with unclear risk in three or more domains assessed by the ROBINS‐I/RoB 2 tool for assessing risk of bias). Subgroup analyses by Omicron subvariants (B.1.1.529/BA.1 sublineages vs. BA.2/BA.4/BA.5 sublineages) and any history of organ transplant were conducted. Data analysis was performed using R (version 4.2.3) and R Studio software (version 2023.03.0). The “*gemtc*” package was used to create a network meta‐analysis model (version 1.0‐1), and the “*rjags*” package (version 4‐14) was used for the Gibbs sampling procedure.

## 3 Results

**FIGURE 1:**
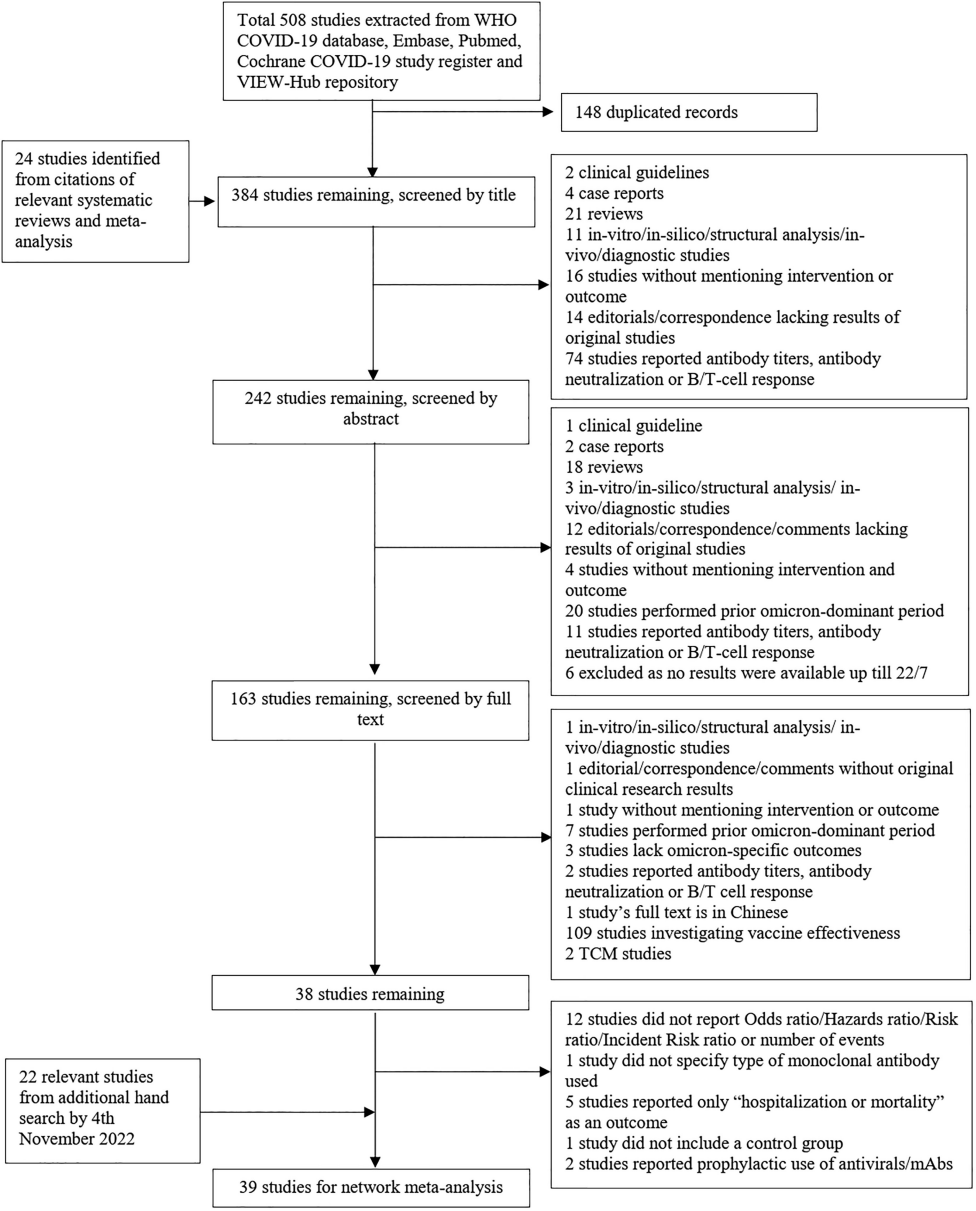
PRISMA flowchart. The identification of studies eligible for inclusion in the current network meta‐analysis. A total of 39 studies were included.

Our search yielded a total of 554 records, of which 39 studies were included, all of which are nonrandomized or observational studies except for a preliminary analysis of molnupiravir use in the PANORAMIC trial (Figure 1). Participants received only usual care or one of the antiviral medications (molnupiravir, nirmatrelvir/ritonavir, and remdesivir) or mAb therapies (sotrovimab and bebtelovimab). Table 1 describes the characteristics of 39 studies eligible for inclusion in the current systematic review and network meta‐analysis. One study (2.6%) was funded or sponsored by pharmaceutical companies [68]. Most of the selected studies were conducted in the United States (15, 38.5%) [33, 39, 40, 42, 45, 46, 47, 48, 52, 57, 59, 62, 67, 68, 70], followed by Hong Kong, China (4, 10.3%) [34, 35, 49, 50], the United Kingdom (4, 10.3%) [19, 36, 53, 63], and Israel (3, 7.7%) [37, 56, 60], with some being conducted in China [54, 58], Canada [43, 66], and Spain (2 each, 5.1%) [64, 65] and the remaining in France [44], South Korea [51], Poland [38], Australia [41], Mexico [55], Italy [61], and Japan (1 each, 2.6%) [69]. Twenty‐seven studies (69.2%) specified the prevalent Omicron subvariant. The majority of studies were conducted during the predominance of Omicron B.1.1.529/BA.1 subvariant (14, 35.9%), followed by BA.2 subvariant (8, 20.5%), B.1.1.529/BA.1 or BA.2 subvariants (4, 10.3%), and BA.4 or BA.5 subvariants (1, 2.6%). Risk of bias analysis for observational studies (*n* = 38) showed that 13 (34.2%) studies were at critical risk of bias, 5 (13.2%) studies at serious risk of bias, and 20 (52.6%) studies at moderate risk of bias (Figure S1). Risk of bias analysis for the single RCT included (i.e., the PANORAMIC trial) was judged to raise some concerns overall (Figure S1).

**TABLE 1:** Characteristics of studies eligible for systematic review and network meta‐analysis.

Author's last name (country)	Study design	Omicron subvariant	Observation period	Age of study population	Special population	Setting	Antivirals or mAbs used	Dosing regimen	Treatment initiation	Disease severity	COVID‐19 vaccination status	Sample size	Outcome (s)
Dryden‐Peterson et al. (US) [33]	Retrospective cohort study	BA.1.1, BA.2, and BA.2.12.1	January 1 to May 15, 2022; January 1 to May 29, 2022, for any subsequent hospitalizations; January 1 to June 12, 2022, for deaths	Aged 50 or above	Nil	Outpatient	Nirmatrelvir/ritonavir	Not specified	Not specified	Not specified	Nirmatrelvir/ritonavir: 76.6% boosted, 17.4% vaccinated, 1.6% partially vaccinated, 4.5% unvaccinated	Nirmatrelvir/ritonavir: *n* = 6036; control: *n* = 24,286	Hospitalization within 14 days of COVID‐19 diagnosis; death within 28 days of COVID‐19 diagnosis
											Control: 48.5% boosted, 37.1% vaccinated, 2.9% partially vaccinated, 11.5% unvaccinated		
Wong et al. (HK, China) [34]	Retrospective cohort study	BA.2.2	February 26 to July 3, 2022	Aged 18 or above	Nil	Outpatient	Molnupiravir	800 mg twice daily for 5 days	Within 5 days of symptom onset	Mild‐to‐moderate	16.1% fully vaccinated	Molnupiravir: *n* = 4983; matched control: *n* = 49,234	All‐cause mortality; hospital admission due to COVID‐19; composite outcome of in‐hospital disease progression (in‐hospital mortality, invasive mechanical ventilation, or intensive care unit admission) and individual in‐hospital outcomes
							Nirmatrelvir/ritonavir	Nirmatrelvir 300 mg and ritonavir 100 mg twice daily for 5 days			33.4% fully vaccinated	Nirmatrelvir/ritonavir: *n* = 5542; matched control: *n* = 54,672	
Wong et al. (HK, China) [35]	Retrospective cohort study	BA.2.2	February 26 to May 3, 2022	Aged 18 or above	Nil	Inpatient	Molnupiravir	800 mg twice daily for 5 days	Within 5 days of symptom onset	Non‐oxygen‐dependent	6.2% fully vaccinated	Molnupiravir: *n* = 1880; matched control: *n* = 1880	All‐cause mortality; composite outcome of disease progression (all‐cause mortality, initiation of invasive mechanical ventilation, intensive care unit admission, or need for oxygen therapy) and individual disease progression outcomes; time to reaching a low viral burden (*Ct* ≥ 30)
							Nirmatrelvir/ritonavir	Nirmatrelvir 300 mg and ritonavir 100 mg twice daily for 5 days			10.5% fully vaccinated	Nirmatrelvir/ritonavir: *n* = 890; matched control: *n* = 890	
Gleeson et al. (UK) [36]	Retrospective cohort study	B.1.1.529	December 17, 2021, to March 31, 2022	Not specified	Kidney transplant recipients	Outpatient	Molnupiravir	Not specified	Within 5 days of RT‐PCR testing	Not specified	Molnupiravir: 9.5% unvaccinated; 14.3% two doses; 66.7% three doses; 9.5% four doses	Molnupiravir: *n* = 21; sotrovimab: *n* = 47; control: *n* = 48	Hospitalization; death; renal and allograft outcomes
											Sotrovimab: 2.1% two doses; 59.6% three doses; 38.3% four doses		
							Sotrovimab	A single dose of 500 mg			Control: 2.1% one dose; 18.8% two doses; 68.7% three doses; 10.4% four doses		
Arbel et al. (Israel) [37]	Retrospective cohort study	B.1.1.529	January 9 to March 10, 2022	Aged 40 or above	High‐risk patients	Outpatient	Nirmatrelvir/ritonavir	Not specified	Not specified	Mild‐to‐moderate	Not specified	Nirmatrelvir/ritonavir: *n* = 3902; control: *n* = 105,352	Hospitalization due to COVID‐19; death due to COVID‐19
Flisiak et al. (Poland) [38]	Retrospective cohort study	B.1.1.529	January 1 to April 30, 2022	Aged 18 or above	Nil	Inpatient	Molnupiravir	800 mg twice daily for 5 days	Not specified	Not specified	Not specified	Molnupiravir: *n* = 203; control: *n* = 387	28‐day mortality; need for mechanical ventilation; clinical improvement
Radcliffe et al. (US) [39]	Retrospective cohort study	B.1.1.529	January 1 to February 16, 2022	Aged 18 or above	Solid organ transplant recipients	Outpatient	Molnupiravir, nirmatrelvir/ritonavir, sotrovimab	Not specified	Within 5 days of symptom onset or the first positive SARS‐CoV‐2 test	Mild‐to‐moderate	87% ≥ 1 dose; 48% ≥ 3 doses of mRNA vaccine or 2 doses of Ad26.COV2.S; 0.8% four doses of mRNA vaccine	Molnupiravir: *n* = 49; nirmatrelvir/ritonavir: *n* = 1; sotrovimab: *n* = 24; control: *n* = 48	Emergency department visits, hospital admission, intensive care unit admission, and death within 30 days after therapy
Ganatra et al. (US) [40]	Retrospective cohort study	B.1.1.529	December 1, 2021, to April 18, 2022	Aged 18 or above	Nil	Outpatient	Nirmatrelvir/ritonavir	Not specified	Within 5 days of COVID‐19 diagnosis	Mild‐to‐moderate	All totally vaccinated	Nirmatrelvir/ritonavir: *n* = 1130; matched control: *n* = 1130	Composite outcome of all‐cause emergency room visit, hospitalization, or death at a 30‐day follow‐up (and their individual outcomes); multisystem symptoms; COVID‐19‐related complications; diagnostic test utilization
Wong et al. (Australia) [41]	Retrospective cohort study	B.1.1.529	December 26, 2021, to January 14, 2022	Aged over 12	Kidney and kidney–pancreas transplant recipients	Outpatient: *n* = 18; inpatient: *n* = 23	Sotrovimab	Not specified	Within 36 h of COVID‐19 diagnosis	Not specified	56.1% three doses; 97.6% two doses; 97.6% one dose; 2.4% unvaccinated	Sotrovimab: *n* = 27; control: *n* = 14	Hospitalization within 30 days of diagnosis; respiratory and multiorgan failure; acute kidney injury; intensive care unit admission; superimposed infections; SARS‐CoV‐2 seroconversion; 30‐day mortality
Aggarwal et al. (US) [42]	Observational cohort study	BA.1 predominant period	December 26, 2021, to March 10, 2022	Aged 18 or above	Nil	Outpatient	Sotrovimab	Not specified	Not specified	Not specified	Sotrovimab: 21.7% unvaccinated; 4.4% one dose; 18.4% two doses; 55.4% ≥ 3 doses	Sotrovimab: *n* = 1542; matched control: *n* = 3663	28‐day all‐cause hospitalization; 28‐day all‐cause mortality; 28‐day emergency department visit
											Control: 24.5% unvaccinated; 5.0% one dose; 19.8% two doses; 50.7% ≥ 3 doses		
Solera et al. (Canada) [43]	Prospective cohort study	BA.1 predominant period	December 15, 2021, to January 24, 2022	Aged 18 or above	Organ transplant recipients	Outpatient	Sotrovimab	Not specified	Within 7 days of symptom onset	Not specified	30.2% two doses; 65.3% ≥ 3 doses	Sotrovimab: *n* = 108; control: *n* = 192	Within 30 days of COVID‐19 diagnosis:need for supplemental oxygen; hospitalization >24 h related to COVID‐19; intensive care unit admission; mechanical ventilation; all‐cause mortality
Chavarot et al. (France) [44]	Retrospective cohort study	B.1.1.529	January 14 to February 13, 2022	Median age of 54 years	Kidney transplant recipients	Outpatient	Sotrovimab	A single dose of 500 mg	Median (IQR) of 5 (3–9) days since symptom onset	Mild‐to‐moderate	Sotrovimab: 4.0% one dose; 4.0% two doses; 64.0% three doses; 12.0% four doses	Sotrovimab: *n* = 25; control: *n* = 100	Mortality; severe COVID‐19 (mortality and/or intensive care unit admission)
											Control: 1.1% one dose; 5.3% two doses; 61.1% three doses; 25.3% four doses		
Yetmar et al. (US) [45]	Retrospective cohort study	B.1.1.529	January 1 to May 30, 2022	Aged 18 or above	Solid organ transplant recipients	Outpatient	Bebtelovimab	A single dose of 175 mg	Not specified	Mild‐to‐moderate	Bebtelovimab: 45.7% boosted; 28.3% fully vaccinated; 12.0% partially vaccinated; 14.1% unvaccinated	Bebtelovimab: *n* = 92; sotrovimab: *n* = 269	COVID‐19‐related hospitalization within 30 days of diagnosis; intensive care unit admission; 30‐day mortality
							Sotrovimab	A single dose of 500 mg			Sotrovimab: 4.1% boosted; 62.1% fully vaccinated; 20.8% partially vaccinated; 13.0% unvaccinated		
Hedvat et al. (US) [46]	Retrospective cohort study	BA.1	December 16, 2021, to January 19, 2022	Aged 18 or above	Solid organ transplant recipients	Outpatient	Nirmatrelvir/ritonavir	Not specified	Within 5 days of symptom onset	Mild‐to‐moderate	Nirmatrelvir/ritonavir: 3.6% one dose; 28.6% two doses; 53.6% three doses	Nirmatrelvir/ritonavir: *n* = 28; sotrovimab: *n* = 51; control: *n* = 75	Hospitalization or death from any cause through Day 30; COVID‐19‐related hospitalization or death at 30 days; acute kidney injury; use of supplemental oxygen; receipt of other COVID‐19 treatment; acute allograft rejection episodes through Day 30
							Sotrovimab		Within 10 days of symptom onset		Sotrovimab: 3.9% one dose; 27.5% two doses; 52.9% three doses		
											Control: 4.0% one dose; 29.3% two doses; 52.0% three doses		
Piccicacco et al. (US) [47]	Retrospective cohort study	B.1.1.529	December 27, 2021, to February 4, 2022	Aged 12 or above	High‐risk patients	Outpatient	Remdesivir	200 mg on Day 1, 100 mg on Days 2 and 3	Within 5 days of symptom onset	Mild‐to‐moderate	Remdesivir: 43.9% boosted; 83.0% initial vaccine series completed; 17.0% unvaccinated	Remdesivir: *n* = 82; sotrovimab: *n* = 88; control: *n* = 90	Composite of COVID‐19‐related hospitalization and emergency department visit within 29 days from symptom onset (and their individual outcomes); 29‐day all‐cause mortality; adverse drug events
							Sotrovimab	A single dose of 500 mg	Within 7 days of symptom onset		Sotrovimab: 48.9% boosted; 76.1% initial vaccine series completed; 23.9% unvaccinated		
											Control: 35.6% boosted; 65.6% initial vaccine series completed; 34.4% unvaccinated		
Wang et al. (US) [48]	Retrospective cohort study	Not specified	January 1 to June 8, 2022	Aged 18 or above	Nil	Outpatient	Nirmatrelvir/ritonavir	Not specified	Within 5 days of COVID‐19 diagnosis	Not specified	With documented COVID‐19 vaccine:nirmatrelvir/ritonavir 19.9%; molnupiravir 12.7%	Nirmatrelvir/ritonavir: *n* = 2226; molnupiravir: *n* = 2226 (after 1:1 matching)	COVID‐19 rebound outcomes:COVID‐19 infections, COVID‐19 related symptoms, and hospitalizations
							Molnupiravir						
Yip et al. (HK, China) [49]	Retrospective cohort study	BA.2	February 16 to March 31, 2022	Mean age of 49.2 years	Nil	Outpatient	Nirmatrelvir/ritonavir	Not specified	Within 5 days of symptom onset	Mild‐to‐moderate	Age‐ and sex‐specified complete vaccination rate:nirmatrelvir/ritonavir 42.7%; molnupiravir 36.2%; control 56.1%	Nirmatrelvir/ritonavir: *n* = 4921; molnupiravir: *n* = 4798; control: *n* = 4758 (after weighting)	Hospitalization; composite of intensive care unit admission, invasive mechanical ventilation use, and/or death
							Molnupiravir						
Wai et al. (HK, China) [50]	Retrospective cohort study	BA.2	February 22 to March 31, 2022	Aged 60 or above, or younger patients with at least one chronic disease	High‐risk patients	Outpatient cohort and inpatient cohort	Molnupiravir	Not specified	Within 7 days from the index date	Mild‐to‐moderate	Not specified	Outpatient cohort:molnupiravir *n* = 5345; nirmatrelvir‐ritonavir *n* = 4442; control: *n* = 23,430	28‐day all‐cause mortality; re‐attendance to the designated clinic or subsequent hospital admission through the emergency department within 28 days from index date (outpatient cohort); unplanned readmission through the emergency department within 28 days (inpatient cohort)
							Nirmatrelvir/ritonavir					Inpatient cohort:molnupiravir *n* = 799; nirmatrelvir‐ritonavir *n* = 282; control: *n* = 20,057	
Lim et al. (South Korea) [51]	Retrospective cohort study	Not specified	January 26 to March 31, 2022	Mean age of 68.5 years	On hemodialysis	Inpatient	Remdesivir	100 mg on Day 1, 50 mg for the next 2–4 days	Within 7 days of symptom onset	Moderate or severe	Not specified	Remdesivir: *n* = 44; control: *n* = 74	Composite of in‐hospital mortality, use of a high‐flow nasal cannula, or transfer to intensive care unit; aggravation of disease severity; changes in NEWS during hospitalization
Razonable et al. (US) [52]	Retrospective cohort study	BA.2	March 20 to June 14, 2022	Aged 18 or above	High‐risk patients	Outpatient	Bebtelovimab	A single dose of 175 mg	Within 7 days of symptom onset	Mild‐to‐moderate	Full vaccination status:bebtelovimab 92.6%; nirmatrelvir/ritonavir 92.5%	Bebtelovimab: *n* = 2833; nirmatrelvir/ritonavir *n* = 774	Progression to severe outcome within 30 days; intensive care unit admission by Day 30; 30‐day all‐cause mortality
							Nirmatrelvir/ritonavir	Nirmatrelvir 150 mg or 300 mg and ritonavir 100 mg twice daily for 5 days	Within 5 days of symptom onset				
Brown et al. (UK) [53]	Observational cohort study	B.1.1.529	December 20, 2021, to January 9, 2022	Median age of 51 years	High‐risk patients	Outpatient	Molnupiravir	Not specified	Within 5 days of symptom onset	Not specified	26% ≥ 3 doses; 7% two doses; 1% one dose; 2% unvaccinated; 1% unknown; 62.4% data not available	Molnupiravir: *n* = 442; sotrovimab: *n* = 186; eligible but declined treatment: *n* = 222	Hospitalization for COVID‐19 within 14 days
							Sotrovimab						
Liu et al. (China) [54]	Single‐center, retrospective, observational study	BA.2	March 26 to May 31, 2022	Aged over 60	Nil	Inpatient	Molnupiravir	800 mg twice daily for 5 days	Within 5 days of symptom onset or the first positive nucleic acid test	Molnupiravir: 46.2% mild; 42.3% moderate; 11.5% severe/critical	Molnupiravir: 34.6% fully vaccinated; 61.5% unvaccinated	Molnupiravir: *n* = 26; control: *n* = 16	Composite of viral shedding in nasopharyngeal swabs and disease progression (all‐cause mortality, initiation of oxygen supply through high‐flow device or invasive mechanical ventilation, or intensive care unit admission), and their individual outcomes
										Control: 50.0% mild; 37.5% moderate; 12.5% severe/critical	Control: 18.8% fully vaccinated; 81.3% unvaccinated		
Rajme‐López et al. (Mexico) [55]	Prospective cohort comparative study	BA.1/BA.2	December 1, 2021 to April 30, 2022	Aged 18 or above	High‐risk patients	Outpatient	Remdesivir	200 mg on Day 1, 100 mg on Days 2 and 3	Within 7 days of symptom onset	Mild‐to‐moderate	Complete vaccination status: remdesivir 83.3%; control 76.4%	Remdesivir: *n* = 54; control: *n* = 72	Composite of hospitalization or death from any cause at Day 28 after symptom onset; all‐cause hospitalization; COVID‐19‐related hospitalization; all‐cause death
Najjar‐Debbiny et al. (Israel) [56]	Retrospective cohort study	Not specified	January 1 to February 28, 2022	Aged 18 or above	High‐risk patients	Outpatient	Molnupiravir	Not specified	Within 5 days of the positive SARS‐CoV‐2 test date	Mild‐to‐moderate	Adequate COVID‐19 vaccination:molnupiravir 74.6%; control 76.1%	Molnupiravir: *n* = 2661; matched control: *n* = 2661	Composite of severe COVID‐19 or COVID‐19‐specific mortality, and their individual outcomes
Lewnard et al. (US) [57]	Observational cohort study	BA.4/BA.5	December 31, 2021, to July 29, 2022	Aged 12 or above	Nil	Outpatient	Nirmatrelvir/ritonavir	Not specified	Not specified	Not specified	Nirmatrelvir/ritonavir: 10.3% four doses; 66.9% three doses; 16.0% two doses; 0.6% one dose; 6.1% unvaccinated	Nirmatrelvir/ritonavir: *n* = 4329; matched control: *n* = 20,980	All‐cause hospital admission; acute respiratory infection‐associated hospital admission
											Control: 3.5% four doses; 73.8% three doses; 16.0% two doses; 0.3% one dose; 6.5% unvaccinated		
Cai et al. (China) [58]	Retrospective study	Not specified	April 7 to June 21, 2022	Aged 18 or above	Patients with acute kidney injury	Not specified	Nirmatrelvir/ritonavir	Not specified	Not specified	Not specified	With vaccine:nirmatrelvir/ritonavir 13.1%; control 11.6%	Nirmatrelvir/ritonavir: *n* = 61; control: *n* = 43	Lung infection; cardiovascular disease‐related mortality; all‐cause mortality; noninvasive ventilation; invasive ventilation; intensive care unit; length of hospital stay; time to viral elimination
McCreary et al. (US) [59]	Retrospective cohort study	Not specified	March 30 to May 28, 2022	Aged 12 or above	High‐risk patients	Outpatient	Bebtelovimab	175 mg	Not specified	Mild‐to‐moderate	Not specified	Bebtelovimab: *n* = 930; matched control: *n* = 930	Hospitalization or death at 28 days; 28‐day hospitalization, death, emergency department visit without hospitalization, and the composite outcome of emergency department visit or hospitalization
Arbel et al. (Israel) [60]	Retrospective cohort study	BA.1	January 16 to March 31, 2022	Aged 40 or above	High‐risk patients	Outpatient	Molnupiravir	Not specified	Not specified	Not specified	Not specified	Molnupiravir: *n* = 1069; control: *n* = 18,799	Hospitalization related to COVID‐19; death due to COVID‐19
Gentile et al. (Italy) [61]	Retrospective cohort study	Not specified	February 18 to June 30, 2022	Aged 19 or above	High‐risk patients	Outpatient	Nirmatrelvir/ritonavir	Nirmatrelvir 300 mg and ritonavir 100 mg twice daily for 5 days	Within 5 days of symptom onset	Mild‐to‐moderate	≥2 doses received:nirmatrelvir/ritonavir 98.2%; molnupiravir 94.5%	Nirmatrelvir/ritonavir: *n* = 111; molnupiravir: *n* = 146	COVID‐19‐related and all‐cause hospitalization and mortality
							Molnupiravir	800 mg twice daily for 5 days					
Zheng et al. (UK) [19]	Observational cohort study	BA.1 (Period 1) and BA.2 (Period 2)	Period 1: December 16, 2021, to February 10, 2022 Period 2: February 16 to May 1, 2022	Aged 18 or above	High‐risk patients	Outpatient	Sotrovimab	Not specified	Within 5 days of symptom onset	Not specified	Sotrovimab: 88.4% ≥ 3 doses; 8.0% two doses; 1.7% one dose; 1.9% unvaccinated	Period 1: sotrovimab: *n* = 3331; molnupiravir: *n* = 2689 Period 2: sotrovimab: *n* = 5979; molnupiravir: *n* = 1970	28‐day COVID‐19‐related hospitalization or death; 28‐day all‐cause hospitalization or death; 60‐day COVID‐19‐related hospitalization or death
							Molnupiravir				Molnupiravir: 86.5% ≥ 3 doses; 9.0% two doses; 1.8% one dose; 2.6% unvaccinated		
Qian et al. (US) [62]	Retrospective cohort study	Not specified	January 23 to May 30, 2022	Aged 18 or above	Patients with systemic autoimmune rheumatic diseases	Outpatient	Nirmatrelvir/ritonavir	Not specified	Not specified	Not specified	Nirmatrelvir/ritonavir: 84.0% additional doses; 13.4% two doses mRNA or one dose adenovirus; 0% partially vaccinated; 2.6% unvaccinated	Nirmatrelvir/ritonavir: *n* = 307; control: *n* = 278	Severe COVID‐19 (hospitalization and/or death within 30 days); COVID‐19 rebound
											Control: 73.4% additional doses; 20.1% two doses mRNA or one dose adenovirus; 0% partially vaccinated; 6.5% unvaccinated		
Butler et al. (UK) [63]	Randomized, controlled open‐label, platform adaptive trial (PANORAMIC)	Not specified	December 8, 2021 to April 27, 2022	Aged 18 or above	High‐risk patients	Outpatient	Molnupiravir	800 mg twice daily for 5 days	Within 5 days of symptom onset	Not specified	Molnupiravir: 2% four doses; 92% three doses; 4% two doses; <1% one dose; 1% missing	Molnupiravir: *n* = 12,821; usual care: *n* = 12,962	All‐cause, nonelective hospital admission and/or death within 28 days of randomization
											Usual care: 2% four doses; 93% three doses; 4% two doses; <1% one dose; 1% missing		
Villamarín et al. (Spain) [64]	Prospective cohort study	Not specified	January 1 to April 30, 2022	Aged above 18	Kidney transplant recipients	Outpatient	Molnupiravir	800 mg twice daily for 5 days	Within 5 days of SARS‐CoV‐2 infection diagnosis	Mild	>2 doses:molnupiravir 89%; remdesivir 87%	Molnupiravir: *n* = 9; remdesivir: *n* = 7	Hospital admission
							Remdesivir	200 mg on Day 1, 100 mg on Days 2 and 3					
Cacho et al. (Spain) [65]	Retrospective cohort study	Not specified	November 1, 2021, to February 28, 2022	Mean age of 58 years	Kidney transplant recipients	Outpatient	Remdesivir	200 mg on Day 1, 100 mg daily for two or four more days	Within 10 days of disease onset	Remdesivir: 10.5% asymptomatic; 47.4% mild; 24.6% moderate; 17.5% severe	Remdesivir: 1.7% unvaccinated; 0% one dose; 19.3% two doses; 73.7% three doses; 0% four doses; 5.3% unknown	Remdesivir: *n* = 57; control: *n* = 41	Hospitalization; need for oxygen during hospital stay; pneumonia; severe disease or death; 30‐day death, discharged, or still hospitalized
										Control: 17.1% asymptomatic; 58.6% mild; 12.2% moderate; 12.2% severe	Control: 0% unvaccinated; 4.9% one dose; 9.8% two doses; 68.3% three doses; 7.3% four doses; 9.8% unknown		
Solera et al. (Canada) [66]	Single‐center, prospective cohort study	BA.2	April 1 to May 5, 2022	Aged 18 or above	Organ transplant recipients	Outpatient	Remdesivir	200 mg on Day 1, 100 mg on Days 2 and 3	Within 7 days of symptom onset	Not specified	Remdesivir: 90.7% ≥ 3 doses; 9.3% < 3 doses	Remdesivir: *n* = 86; control: *n* = 106	COVID‐19‐related hospitalization within 30 days; need for supplemental oxygen; admission to intensive care unit; mechanical ventilation; all‐cause mortality
											Control: 89.6% ≥ 3 doses; 10.4% < 3 doses		
Aggarwal et al. (US) [67]	Retrospective cohort study	BA.2/BA.2.12.1	March 26 to June 23, 2022	Aged 18 or above	High‐risk patients	Outpatient	Nirmatrelvir/ritonavir	Not specified	Within 10 days of a positive SARS‐CoV‐2 test	Not specified	Nirmatrelvir/ritonavir: 18.1% unvaccinated; 3.9% one dose; 13.5% two doses; 64.5% ≥ 3 doses	Nirmatrelvir/ritonavir: *n* = 3614; matched control: *n* = 4835	All‐cause hospitalization within 28 days; COVID‐19 specific 28‐day hospitalization; 28‐day all‐cause mortality; 28‐day all‐cause emergency department visit
											Control: 19.7% unvaccinated; 4.2% one dose; 15.1% two doses; 61.0% ≥ 3 doses		
Zhou et al. (US) [68]	Retrospective cohort study	Not specified	December 22, 2021 to June 8, 2022	Aged 12 or above	High‐risk patients	Outpatient	Nirmatrelvir/ritonavir	Not specified	Not specified	Not specified	Receipt of ≥1 COVID‐19 vaccination:nirmatrelvir/ritonavir 67.6%; control: 45.5%	Nirmatrelvir/ritonavir: *n* = 2808; matched control: *n* = 10,849	All‐cause hospitalization within 30 days
Suzuki et al. (Japan) [69]	Retrospective cohort study	Not specified	January to April 2022	Aged 18 or above	High‐risk patients	Inpatient	Molnupiravir	Not specified	Within 5 days of symptom onset	Mild‐to‐moderate	Received vaccine twice or more:molnupiravir 78.3%; control 77.0%	Molnupiravir: *n* = 259; matched control: *n* = 259	Any clinical deterioration; the need for mechanical ventilation; all‐cause death
Razonable et al. (US) [70]	Retrospective cohort study	B.1.1.529 and BA.2	Sotrovimab cohort: January 1 to March 20, 2022 Bebtelovimab cohort: March 21 to April 30, 2022	Aged 18 or above	High‐risk patients	Outpatient	Sotrovimab	A single 500 mg dose	Within 7 days of symptom onset	Mild‐to‐moderate	Sotrovimab cohort: 86.3% primary vaccination; 63.5% booster dose	Sotrovimab cohort: *n* = 2182 Bebtelovimab cohort: *n* = 1690	Progression to severe outcomes within 30 days; intensive care unit admission; 30‐day all‐cause mortality
							Bebtelovimab	A single 175 mg dose			Bebtelovimab cohort: 92.9% primary vaccination; 77.2% booster dose		

**FIGURE 2:**
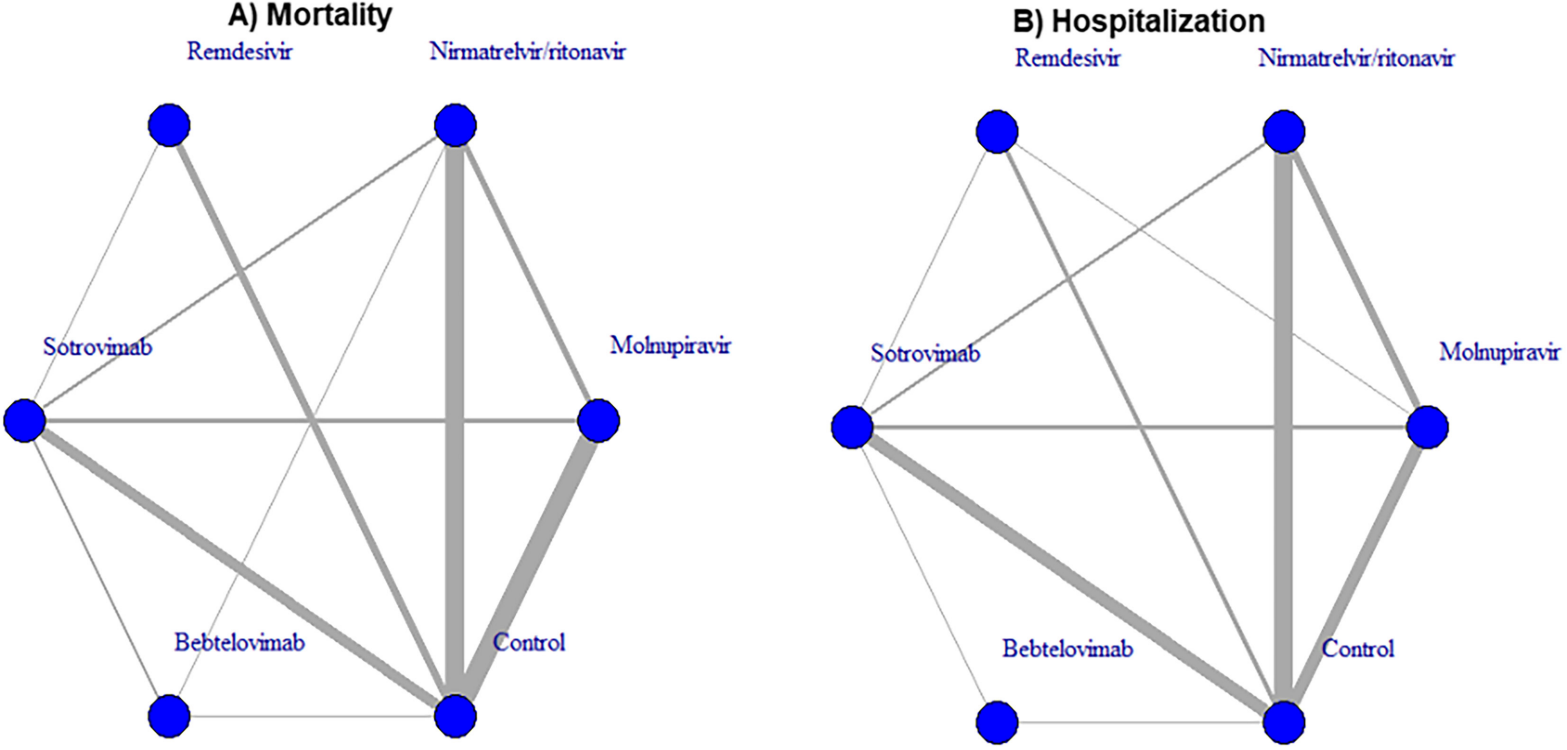
Network diagram of direct comparisons of treatment groups for the outcome of (a) mortality and (b) hospitalization. Lines between two nodes in the network diagram mean there is direct evidence between two treatments; the thickness of the line corresponds to the number of studies in which each direct comparison is made. Here, the network diagram of mortality (a) shows 10 direct comparisons, and the network diagram of hospitalization (b) shows 10 direct comparisons.

**FIGURE 3:**
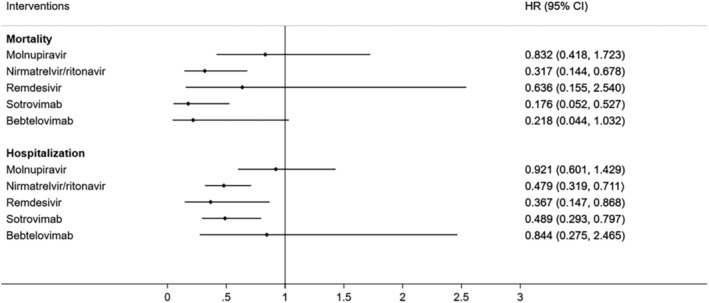
Forest plot of mortality and hospitalization outcomes for comparison between each intervention and control. Associations between each intervention and mortality or hospitalization outcomes are presented in this forest plot. Hazard ratios < 1 imply a lower risk of the study outcomes with treatment use compared to control, where results are considered statistically significant when 95% credible intervals do not include 1 (null effect).

The network diagram displaying direct comparisons between treatment groups for outcomes is shown in Figure 2. A greater number of studies provided direct comparisons between nirmatrelvir/ritonavir and control, molnupiravir and control, and sotrovimab and control (i.e., the thicker the line between treatments, the more studies in which the direct comparison was made). The results of the network meta‐analysis are summarized in Table 2 and Figure 3; 32 (82%) and 27 (69%) studies were evaluated for mortality and hospitalization, respectively. Table 2 provides a league table of all pairwise treatment comparisons, highlighting the most effective treatments. When compared to controls, sotrovimab and nirmatrelvir/ritonavir are associated with lower risks of all‐cause mortality (Table 2A and Figure 3), whereas sotrovimab, remdesivir, and nirmatrelvir/ritonavir are associated with lower risks of all‐cause hospitalization (Table 2B and Figure 3).

**TABLE 2:** League tables with network meta‐analytic estimates for the outcome of (A) mortality and (B) hospitalization.

(A) Mortality					
Molnupiravir	**0.38** **(0.138, 0.988)**	0.762 (0.156, 3.551)	**0.211** **(0.055, 0.695)**	0.261 (0.047, 1.362)	1.201 (0.58, 2.392)
**2.634** **(1.012, 7.248)**	Nirmatrelvir/ ritonavir	2.014 (0.406, 9.881)	0.557 (0.139, 2.025)	0.688 (0.131, 3.591)	**3.156** **(1.474, 6.935)**
1.312 (0.282, 6.43)	0.496 (0.101, 2.465)	Remdesivir	0.276 (0.044, 1.602)	0.344 (0.042, 2.77)	1.573 (0.394, 6.461)
**4.743** **(1.439, 18.139)**	1.796 (0.494, 7.208)	3.618 (0.624, 22.605)	Sotrovimab	1.245 (0.272, 6.002)	**5.678** **(1.897, 19.323)**
3.83 (0.734, 21.261)	1.453 (0.279, 7.633)	2.909 (0.361, 23.836)	0.803 (0.167, 3.681)	Bebtelovimab	4.591 (0.969, 22.527)
0.832 (0.418, 1.723)	**0.317** **(0.144, 0.678)**	0.636 (0.155, 2.54)	**0.176** **(0.052, 0.527)**	0.218 (0.044, 1.032)	Control
(B) Hospitalization					
Molnupiravir	**0.52** **(0.305, 0.867)**	0.398 (0.146, 1.02)	**0.53** **(0.28, 0.975)**	0.917 (0.275, 2.876)	1.086 (0.7, 1.664)
**1.923** **(1.153, 3.275)**	Nirmatrelvir/ ritonavir	0.765 (0.285, 1.965)	1.019 (0.544, 1.887)	1.765 (0.537, 5.501)	**2.087** **(1.407, 3.137)**
2.515 (0.98, 6.854)	1.307 (0.509, 3.511)	Remdesivir	1.335 (0.512, 3.579)	2.31 (0.57, 9.303)	**2.725** **(1.153, 6.824)**
**1.886** **(1.026, 3.571)**	0.981 (0.53, 1.839)	0.749 (0.279, 1.952)	Sotrovimab	1.732 (0.552, 5.263)	**2.047** **(1.255, 3.413)**
1.09 (0.348, 3.639)	0.567 (0.182, 1.862)	0.433 (0.107, 1.755)	0.577 (0.19, 1.813)	Bebtelovimab	1.185 (0.406, 3.631)
0.921 (0.601, 1.429)	**0.479** **(0.319, 0.711)**	**0.367** **(0.147, 0.868)**	**0.489** **(0.293, 0.797)**	0.844 (0.275, 2.465)	Control

*Note:* A total of 32 and 27 studies were included in the analyses for the outcome of (A) mortality and (B) hospitalization, respectively. Comparisons should be read from top to bottom and from left to right. Results are the hazard ratios with a 95% credible interval in the column‐defining therapy compared with the row‐defining therapy. Values in bold are those in which the 95% credible intervals do not include 1 (null effect).

Nirmatrelvir/ritonavir was the only antiviral medication associated with lower risks in both mortality (HR = 0.317, 95%CrI = 0.144–0.678) and hospitalization (HR = 0.479, 95%CrI = 0.319–0.711) when compared to controls (Table 2 and Figure 3). Molnupiravir use was associated with comparable risks of mortality (HR = 0.832, 95%CrI = 0.418–1.723) and hospitalization (HR = 0.921, 95%CrI = 0.601–1.429) to controls. Nirmatrelvir/ritonavir was associated with reduced risks in mortality (HR = 0.38, 95%CrI = 0.138–0.988) and hospitalization (HR = 0.52, 95%CrI = 0.305–0.867) when compared to molnupiravir (Table 2). Remdesivir was not more effective in lowering the mortality risk (HR = 0.636, 95%CrI = 0.155–2.54) than controls yet significantly protective against hospitalization (HR = 0.367, 95%CrI = 0.147–0.868). Regarding the mAb therapy, sotrovimab was associated with lower risks of both mortality (HR = 0.176, 95%CrI = 0.052–0.527) and hospitalization (HR = 0.489, 95%CrI = 0.293–0.797) when compared to controls. Meanwhile, bebtelovimab was associated with comparable risks of mortality (HR = 0.218, 95%CrI = 0.044–1.032) and hospitalization (HR = 0.844, 95%CrI = 0.275–2.465) to controls. Furthermore, sotrovimab was associated with lower risks of mortality (HR = 0.211, 95%CrI = 0.055–0.695) and hospitalization (HR = 0.53, 95%CrI = 0.28–0.975) when compared to molnupiravir (Table 2). Our statistical test showed no evidence of publication bias (Egger's test *p* = 0.243). Results of sensitivity analyses (Table S2) were broadly consistent with primary results.

The results of subgroup analyses are presented in Supplementary Table S3. Stratified by Omicron subvariants, sotrovimab was effective in reducing death (HR = 0.146, 95%CrI = 0.025–0.518) and hospitalization (HR = 0.485, 95%CrI = 0.272–0.826) against B.1.1.529/BA.1 sublineage than controls, whereas nirmatrelvir/ritonavir reduced the risks of death (HR = 0.147, 95%CrI = 0.026–0.821) and hospitalization (HR = 0.744, 95%CrI = 0.585–0.978) against BA.2/BA.4/BA.5 sublineages. For organ transplant patients infected with the Omicron variant (10 studies), both sotrovimab (HR = 0.316, 95%CrI = 0.152–0.551) and remdesivir were effective in lowering the risk of hospitalization (HR = 0.191, 95%CrI = 0.029–0.874) when compared to controls. Among nonorgan transplant recipients (29 studies), nirmatrelvir/ritonavir appeared to be superior to controls (mortality: HR = 0.299, 95%CrI = 0.124–0.691; hospitalization: HR = 0.48, 95%CrI = 0.304–0.745) or molnupiravir (mortality: HR = 0.344, 95%CrI = 0.11–0.994; hospitalization: HR = 0.522, 95%CrI = 0.285–0.933) in the prevention of severe outcomes. A summary of the key findings from our systematic review and network meta‐analysis is presented in Table 3.

**TABLE 3:** Summary of key findings.

Key findings
‐ In this systematic review and network meta‐analysis, nirmatrelvir/ritonavir and sotrovimab are associated with a probable reduction in mortality risk for patients infected with the Omicron variant of SARS‐CoV‐2. ‐ The use of nirmatrelvir/ritonavir, remdesivir, or sotrovimab may also lower the risk of hospitalization for these patients. ‐ Sotrovimab appears effective against the B.1.1.529/BA.1 Omicron subvariant, whereas nirmatrelvir/ritonavir is likely effective against the BA.2/BA.4/BA.5 subvariant. ‐ Sotrovimab and remdesivir have also shown effectiveness in reducing the risks of hospitalization specifically among organ transplant recipients affected by the Omicron variant.

## 4 Discussion

Among the antivirals and mAb therapies investigated in this systematic review and network meta‐analysis, the use of nirmatrelvir/ritonavir or sotrovimab probably reduces the mortality risk of patients with SARS‐CoV‐2 infection of the Omicron variant, whereas the use of nirmatrelvir/ritonavir, remdesivir or sotrovimab may also lower their risk of hospitalization. When comparing therapies, nirmatrelvir/ritonavir and sotrovimab appear to be more effective than molnupiravir in reducing the risk of disease progression to hospitalization or death. Notably, sotrovimab is likely effective against B.1.1.529/BA.1 sublineage, and nirmatrelvir/ritonavir against BA.2/BA.4/BA.5 sublineages. Both sotrovimab and remdesivir also reduce the risk of hospitalization among organ transplant recipients affected by the Omicron variant. In view of the paucity of data about the clinical use of bebtelovimab in COVID‐19 patients during the Omicron wave, no conclusive evidence on its indications can be made in the current study. Besides, the generally high risk of bias and heterogeneity of the included studies should be acknowledged.

Overall, our results are generally consistent with those observed in previous systematic reviews and network meta‐analyses of RCTs of drug treatments for COVID‐19, namely, a reduced mortality risk with nirmatrelvir/ritonavir use and significantly fewer hospital admissions among COVID‐19 patients on nirmatrelvir/ritonavir or remdesivir [20, 21, 23]. It has also been demonstrated that nirmatrelvir/ritonavir will likely be more effective than molnupiravir in preventing hospitalization [21]. As this evidence was generated from studies conducted before the Omicron wave, it is reassuring that both nirmatrelvir/ritonavir and remdesivir appear to have maintained their clinical effectiveness against the Omicron variant, as previously suggested by their equipotent antiviral activity observed across SARS‐CoV‐2 VOC in vitro [4, 71]. Although molnupiravir has also been shown to offer some benefit in lowering the risk of mortality [20, 21, 23], its effect in patients with Omicron infection might only be evident in certain patient subgroups, such as the elderly [60], who have not been adequately vaccinated [34], and those requiring hospitalization for COVID‐19 [35, 38].

Regarding the use of mAb therapy in COVID‐19 patients, caution is needed to determine and apply specific mAb products that are susceptible to the evolving variants and subvariants of SARS‐CoV‐2, thus the clinical effectiveness of mAb therapies should always be evaluated in light of particular viral strains. Consistent with previous evidence suggesting fewer hospital admissions among patients with nonsevere COVID‐19 on mAb therapies in the pre‐Omicron era [72], our results conclude that sotrovimab use reduces both hospitalization and mortality risks when directed against B.1.1.529/BA.1 but not BA.2 or subsequent Omicron subvariants. These are in line with in vitro data that find a median of four‐fold reduction in neutralizing activity of sotrovimab against BA.1, and a 17‐fold reduction against BA.2, alongside a complete loss of activity against BA.2.12.1/BA.4/BA.5 subvariants [4, 9]. Based on in vitro data showing the retention of neutralizing activity of bebtelovimab against most Omicron subvariants [4, 9], and limited evidence from a phase 2 clinical trial, bebtelovimab has been granted the EUA for treating nonhospitalized patients with mild‐to‐moderate COVID‐19 [73]. Nevertheless, only three observational studies on bebtelovimab against Omicron infection were eligible for inclusion in the current review, and recent experimental evidence has suggested potential resistance of Omicron BQ.1 and BQ.1.1 subvariants to this mAb therapy [73]. In view of the replacement with BQ.1 and BQ.1.1 and, subsequently, XBB and XBB.1.5 subvariants that bebtelovimab has demonstrated no efficacy against in vitro [16, 74], it is no longer authorized for emergency use [75], and at the time of writing, no anti‐SARS‐CoV‐2 mAb therapies are recommended for treating COVID‐19 [76].

Except for five studies that explored the real‐world effectiveness of oral antivirals among hospitalized COVID‐19 patients with Omicron infection, the remaining studies were conducted in the outpatient setting. Given the relatively low baseline risk of Omicron infection, most studies aimed to examine the effect of antivirals and mAb therapies in preventing patients from progressing to severe COVID‐19 requiring hospitalization and/or death. Accordingly, the population of interest for these drug therapies was mostly patients with mild‐to‐moderate COVID‐19, who were also at higher risk of progression to severe disease. Apart from old age and obesity, risk factors for developing severe COVID‐19 include chronic comorbidities (such as hypertension; diabetes; heart conditions; chronic lung, kidney, or liver diseases; and malignancy), solid organ or stem cell transplant, immunosuppressant use, and incomplete vaccination status [77]. Notably, the definition of complete vaccination against COVID‐19 varied across studies by vaccine type (i.e., the number of doses constituting the primary series, or conferring sufficient protection), with or without boosters, patient characteristics (viz., any history of organ transplant), and time since last vaccine dose. In some studies, such information was either lacking or not adequately adjusted for at baseline, and some intervention groups were likely to have been vaccinated with more doses than others. Consequently, subgroup analyses of drug treatment effects by vaccination status could not be evaluated in the current review. Furthermore, only a small proportion of the included studies reported drug effectiveness by different age groups; hence, subgroup analyses by age could not be examined in the current results.

Taking clinical effectiveness and ease of administration into account, our findings support current guidelines that recommend the early initiation of nirmatrelvir/ritonavir over intravenous remdesivir for nonhospitalized patients with mild‐to‐moderate COVID‐19 at high risk of progression to severe disease (leading to hospitalization or death) and considering molnupiravir (with limited efficacy) as an alternative therapy only when neither of the previous two is accessible or clinically appropriate [76, 78, 79]. Since April 5, 2022, the EUA for sotrovimab has been revoked in view of the growing case incidence of Omicron BA.2 infection, of which this mAb therapy is likely ineffective [80]; bebtelovimab is also no longer authorized for emergency use from November 30, 2022, considering the predominance of BQ.1 and BQ.1.1 subvariants at that time [75]. Therefore, the scientific and healthcare communities should remain vigilant about the evolving viral mutations and rapid emergence of SARS‐CoV‐2 (sub)variants that may have occurred naturally, or induced by the positive selection of those that bear resistance to specific mAb therapies [8]. Furthermore, the immunity status of COVID‐19 patients, whether acquired through previous infection or vaccination, may also play a role in the clinical efficacy and safety of mAb therapies. This should be taken into account along with viral sequencing to minimize treatment failure, especially when new candidates of anti‐SARS‐CoV‐2 mAb therapies become available in the near future [8, 72].

Beyond the clinical effectiveness of antivirals and mAb therapies, there have been several concerns regarding their safety and accessibility. Firstly, there has been an increasing number of reports on the development of “COVID‐19 rebound” following oral antiviral treatment, especially nirmatrelvir/ritonavir, in patients with Omicron infection, which is characterized by the recurrence of COVID‐19‐related symptoms and/or re‐positive viral test results (with high viral load and/or culturable virus) 2–8 days after initial recovery [81, 82]. Interestingly, such a rebound phenomenon has been observed in COVID‐19 patients regardless of their vaccination status, and in both immunocompetent patients and organ transplant recipients [48, 81, 83]. Although it has not been associated with viral mutations or reinfection of a different (sub)variant [82, 84], further research is needed to ascertain the risk factors and clinical outcomes of such rebound; and determine if it is related to the natural biphasic pattern of SARS‐CoV‐2 infection, resumption of viral replication after early antiviral treatment, dosage and duration of therapy, individual pharmacokinetics or comorbidities of patients [48, 85, 86].

Secondly, the long‐term safety of antivirals and mAb therapies remain to be investigated, for instance, genetic toxicity and carcinogenicity of molnupiravir in humans, hepatotoxicity of drugs, and the rate of emergency of viral resistance or new variants [79]. Clinicians should also be aware of the potentially significant drug–drug interactions of nirmatrelvir/ritonavir with other concomitant medications [87] and selective pressure and treatment‐emergent resistance of SARS‐CoV‐2 to existing therapies and avoid any inappropriate use of mAb therapies against resistant (sub)variants (for instance, the use of bebtelovimab against BQ.1 and BQ.1.1, or XBB and XBB.1.5 subvariants) [8, 16, 74]. Thirdly, early initiation of drugs can be affected by their limited supplies and accessibility, the feasibility of parenteral administration, and the extensive time and resources needed for viral sequencing to identify the appropriate mAb therapy, if any, for individual patients [8]. Ideally, an evidence‐based scoring system, such as those developed for prioritizing mAb therapies to high‐risk patients [88], will facilitate the optimal distribution of drugs to those who would benefit the most [89].

## 5 Limitations

Several limitations should be acknowledged. Firstly, the reliance on predominantly observational studies with varying degrees of bias and confounding limits the strength of causal inferences about the effectiveness of antivirals and mAb therapies. Secondly, there is considerable heterogeneity among studies due to differences in study populations, follow‐up lengths, treatment duration, timing of treatment initiation, and inconsistent adjustments for factors like vaccination status and comorbidities. Thirdly, our findings are based on data available as of the last search and may be impacted by changes in mortality risk due to immune evasion by emerging variants, underscoring the need for ongoing randomized trials across different variants and patient populations. Finally, despite conducting sensitivity analyses that excluded studies with a critical risk of bias, residual confounding remains a concern.

## 6 Future Research

Further research is needed to address these limitations and enhance our understanding of antiviral and mAb therapies in the context of COVID‐19. Recognizing the absence of safety and efficacy data for treating Omicron infection, ongoing trials (viz., RECOVERY and PANORAMIC) [90, 91] are evaluating currently available therapies to inform the clinical management of COVID‐19 patients. Another research gap involves the safety and efficacy of drug combination therapies (i.e., mAb cocktail or mAb plus antiviral) in improving patient outcomes and reducing the likelihood of treatment‐emergent resistance [8, 21, 92], especially in immunocompromised patients who often experience persistent positivity, virologic failure, and hence an increased chance for escape mutations to emerge [92, 93]. Further research should also focus on head‐to‐head comparisons of therapies and their effects on specific subpopulations, including pediatric patients, pregnant and breastfeeding women, and individuals with varying immunity status. Additionally, research should explore how different SARS‐CoV‐2 subvariants influence treatment outcomes and the potential effects on long COVID. Active pharmacovigilance programs will monitor the long‐term safety of COVID‐19 treatments, whereas genomic sequencing of SARS‐CoV‐2 and preclinical studies will inform the immune evasion and any potential resistance to existing therapies [15]. Moreover, cost‐effectiveness analyses are called for to compare the currently available treatments, as the numbers needed to treat to prevent a COVID‐19‐related hospitalization or death have likely increased since those estimated from trials conducted in the pre‐Omicron era, given the lower baseline risk of Omicron infection and a rising level of population immunity (from vaccination and/or previous infection) [92].

In conclusion, nirmatrelvir/ritonavir or sotrovimab use is associated with mortality benefit in COVID‐19 patients during the Omicron wave, where nirmatrelvir/ritonavir, remdesivir or sotrovimab users may also experience a lower risk of hospitalization. Notably, sotrovimab is effective against B.1.1.529/BA.1 subvariant and nirmatrelvir/ritonavir against BA.2/BA.4/BA.5 subvariants. Evidence on the effectiveness of bebtelovimab against Omicron infection remains scarce. In view of the generally high risk of bias and heterogeneity of the included studies, further evaluation of the growing literature (especially the inclusion of data from ongoing RCTs) is needed to review the recommendations on COVID‐19 treatments. Constant evaluation of the susceptibility of SARS‐CoV‐2 (sub)variants to novel antivirals and mAb therapies is essential to minimize the inappropriate use of therapies against resistant variants, and the feasibility, safety, and efficacy of drug combination therapies should be explored.

## Author Contributions

**Kristy T. K. Lau:** data curation, investigation, visualization, writing – original draft. **Xi Xiong:** data curation, formal analysis, investigation, methodology, visualization, writing – original draft. **Carlos K. H. Wong:** conceptualization, data curation, investigation, methodology, project administration, resources, software, supervision, validation, visualization, writing – original draft. **Ivan C. H. Au:** data curation, formal analysis, methodology. **Angel Y. C. Lui:** data curation, investigation, visualization, writing – original draft. **Gavin Y. T. Tsai:** data curation, investigation, visualization, writing – original draft. **Tingting Wu:** data curation, formal analysis, investigation, visualization. **Lanlan Li:** data curation, investigation. **Eric H.Y. Lau:** project administration, supervision, visualization. **Benjamin J. Cowling:** project administration, supervision, visualization. **Gabriel M, Leung:** project administration, supervision, visualization.

## Conflicts of Interest

B.J.C. has provided scientific advice to Pfizer and AstraZeneca on issues related to disease burden and vaccine effectiveness. He has not provided scientific advice to either company related to COVID‐19 antiviral effectiveness, and he has not received any funding from Pfizer or AstraZeneca for any research on antiviral effectiveness including the current work. All other authors declare no competing interests.

## Dissemination to Participants and Related Patients and Public Communities

We will disseminate our findings to clinician and patient organizations and through traditional media and social media outlets. Study results will be presented at a variety of national and international conferences and forums.

## Supporting information


**Appendix S1** Search strategies.

**Appendix S2** COVID drug NMA R code.

**Figure S1** Risk of bias assessment of observational studies using the ROBINS‐I and randomized controlled trial using the RoB 2.
**Figure S2** Results for hospitalization from node‐splitting approach.
**Table S1** Number of events and effect estimates for the outcome of a) mortality and b) hospitalization in eligible studies.
**Table S2.** League tables with network meta‐analytic estimates for the outcome of a) mortality and b) hospitalization in sensitivity analyses.
**Table S3** League tables with network meta‐analytic estimates for the outcome of a) mortality and b) hospitalization in patient subgroups of 1) B.1.1.529 or BA.1 infection, 2) infection with other Omicron subvariants, 3) organ transplant recipients, and 4) non‐organ transplant recipients.

**Data S1** Supplementary Information.

## Data Availability

Requests for data sharing should be sent to the corresponding author: Carlos K.H. Wong (carlosho@hku.hk). The R codes for network meta‐analysis are available in Appendix S2. The lead author (Carlos K. H. Wong) affirms that the manuscript is an honest, accurate, and transparent account of the study being reported; that no important aspects of the study have been omitted; and that any discrepancies from the study as originally planned (and, if relevant, registered) have been explained.
