# Supplementary material for: Comparative Effectiveness of Antivirals and Monoclonal Antibodies for Treating COVID‐19 Patients Infected With Omicron Variant: A Systematic Review and Network Meta‐Analysis
Source: Influenza Other Respir Viruses. 2024 Dec 25;18(12):e70065. doi: 10.1111/irv.70065 (PMC11669747; doi:10.1111/irv.70065)
Supplement: Supplementary file 1 — Appendix S1 Search strategies. [file IRV-18-e70065-s002.docx]

**Appendix 1**

**Search strategies**

| **PubMed** | \| **Search number** \| **Query** \| \| --- \| --- \| \| **10** \| (((((((((((((((Sotrovimab) OR (Casirivimab)) OR (Imdevimab)) OR (Bamlanivimab)) OR (AZD7442)) ) OR (Ivermectin)) OR (Fluvoxamine) AND (2021/11/24:3000/12/12[pdat])) OR ("sarilumab" or "tocilizumab" or "siltuximab" or "Baricitinib" or "Ruxolitinib" or "Tofacitinib" or "Hydroxychloroquine" or "Interferon" AND (2021/11/24:3000/12/12[pdat]))) OR (((("dexamethasone") OR ("hydrocortisone")) OR ("prednisolone")) OR ("prednisone") AND (2021/11/24:3000/12/12[pdat]))) OR (((((((((((("PF-07321332") OR ("Nirmatrelvir/ritonavir")) OR ("Paxlovid")) OR (Remdesivir)) OR (EIDD-2801)) OR (Lagevrio)) OR (Molnupiravir)) OR (Oseltamivir)) OR (Favipravir)) OR (arbidol hydrochloride)) OR (Ribavirin)) OR (azithromycin) AND (2021/11/24:3000/12/12[pdat]))) OR (((((((((ChAdOx1-S) OR (BNT162b2)) OR (CoronaVac)) OR (BBIBP-CorV)) OR (mRNA-1273)) OR (Ad26.COV2.S)) OR ("NVX-CoV2373")) OR ("Ad5-based COVID-19 vaccine")) OR ("Gam-COVID-Vac") AND (2021/11/24:3000/12/12[pdat])) AND (2021/11/24:3000/12/12[pdat])) AND ((((((((((((((Mortality) OR (Mechanical ventilation)) OR (Hospital admission)) OR (Time to symptom resolution)) OR (Viral clearance)) OR (Time to clinical improvement)) OR (Duration of Hospitalization)) OR (Time to viral clearance)) OR (Symptomatic infection)) OR (ICU admission)) OR (Clinical recovery)) OR (Progression to severe disease)) OR (Discharge rates)) OR (COVID-19 infection) AND (2021/11/24:3000/12/12[pdat]))) AND (((((((("omicron") OR ("B.1.1.529")) OR ("BA.1")) OR ("BA.1.1")) OR ("BA.2")) OR ("BA.2.12.1")) OR ("BA.4")) OR ("BA.5") AND (2021/11/24:3000/12/12[pdat]))) AND (Therapy/Broad[filter]) \| \| **9** \| ((((((((((((((Sotrovimab) OR (Casirivimab)) OR (Imdevimab)) OR (Bamlanivimab)) OR (AZD7442)) ) OR (Ivermectin)) OR (Fluvoxamine) AND (2021/11/24:3000/12/12[pdat])) OR ("sarilumab" or "tocilizumab" or "siltuximab" or "Baricitinib" or "Ruxolitinib" or "Tofacitinib" or "Hydroxychloroquine" or "Interferon" AND (2021/11/24:3000/12/12[pdat]))) OR (((("dexamethasone") OR ("hydrocortisone")) OR ("prednisolone")) OR ("prednisone") AND (2021/11/24:3000/12/12[pdat]))) OR (((((((((((("PF-07321332") OR ("Nirmatrelvir/ritonavir")) OR ("Paxlovid")) OR (Remdesivir)) OR (EIDD-2801)) OR (Lagevrio)) OR (Molnupiravir)) OR (Oseltamivir)) OR (Favipravir)) OR (arbidol hydrochloride)) OR (Ribavirin)) OR (azithromycin) AND (2021/11/24:3000/12/12[pdat]))) OR (((((((((ChAdOx1-S) OR (BNT162b2)) OR (CoronaVac)) OR (BBIBP-CorV)) OR (mRNA-1273)) OR (Ad26.COV2.S)) OR ("NVX-CoV2373")) OR ("Ad5-based COVID-19 vaccine")) OR ("Gam-COVID-Vac") AND (2021/11/24:3000/12/12[pdat])) AND (2021/11/24:3000/12/12[pdat])) AND ((((((((((((((Mortality) OR (Mechanical ventilation)) OR (Hospital admission)) OR (Time to symptom resolution)) OR (Viral clearance)) OR (Time to clinical improvement)) OR (Duration of Hospitalization)) OR (Time to viral clearance)) OR (Symptomatic infection)) OR (ICU admission)) OR (Clinical recovery)) OR (Progression to severe disease)) OR (Discharge rates)) OR (COVID-19 infection) AND (2021/11/24:3000/12/12[pdat]))) AND (((((((("omicron") OR ("B.1.1.529")) OR ("BA.1")) OR ("BA.1.1")) OR ("BA.2")) OR ("BA.2.12.1")) OR ("BA.4")) OR ("BA.5") AND (2021/11/24:3000/12/12[pdat])) \| \| **8** \| ((((((((((((Sotrovimab) OR (Casirivimab)) OR (Imdevimab)) OR (Bamlanivimab)) OR (AZD7442)) ) OR (Ivermectin)) OR (Fluvoxamine) AND (2021/11/24:3000/12/12[pdat])) OR ("sarilumab" or "tocilizumab" or "siltuximab" or "Baricitinib" or "Ruxolitinib" or "Tofacitinib" or "Hydroxychloroquine" or "Interferon" AND (2021/11/24:3000/12/12[pdat]))) OR (((("dexamethasone") OR ("hydrocortisone")) OR ("prednisolone")) OR ("prednisone") AND (2021/11/24:3000/12/12[pdat]))) OR (((((((((((("PF-07321332") OR ("Nirmatrelvir/ritonavir")) OR ("Paxlovid")) OR (Remdesivir)) OR (EIDD-2801)) OR (Lagevrio)) OR (Molnupiravir)) OR (Oseltamivir)) OR (Favipravir)) OR (arbidol hydrochloride)) OR (Ribavirin)) OR (azithromycin) AND (2021/11/24:3000/12/12[pdat]))) OR (((((((((ChAdOx1-S) OR (BNT162b2)) OR (CoronaVac)) OR (BBIBP-CorV)) OR (mRNA-1273)) OR (Ad26.COV2.S)) OR ("NVX-CoV2373")) OR ("Ad5-based COVID-19 vaccine")) OR ("Gam-COVID-Vac") AND (2021/11/24:3000/12/12[pdat])) \| \| **7** \| (((((((((((((Mortality) OR (Mechanical ventilation)) OR (Hospital admission)) OR (Time to symptom resolution)) OR (Viral clearance)) OR (Time to clinical improvement)) OR (Duration of Hospitalization)) OR (Time to viral clearance)) OR (Symptomatic infection)) OR (ICU admission)) OR (Clinical recovery)) OR (Progression to severe disease)) OR (Discharge rates)) OR (COVID-19 infection) \| \| **6** \| ((((((((Sotrovimab) OR (Casirivimab)) OR (Imdevimab)) OR (Bamlanivimab)) OR (AZD7442)) ) OR (Ivermectin)) OR (Fluvoxamine) \| \| **5** \| "sarilumab" or "tocilizumab" or "siltuximab" or "Baricitinib" or "Ruxolitinib" or "Tofacitinib" or "Hydroxychloroquine" or "Interferon" \| \| **4** \| ((("dexamethasone") OR ("hydrocortisone")) OR ("prednisolone")) OR ("prednisone") \| \| **3** \| ((((((((((("PF-07321332") OR ("Nirmatrelvir/ritonavir")) OR ("Paxlovid")) OR (Remdesivir)) OR (EIDD-2801)) OR (Lagevrio)) OR (Molnupiravir)) OR (Oseltamivir)) OR (Favipravir)) OR (arbidol hydrochloride)) OR (Ribavirin)) OR (azithromycin) \| \| **2** \| ((((((((ChAdOx1-S) OR (BNT162b2)) OR (CoronaVac)) OR (BBIBP-CorV)) OR (mRNA-1273)) OR (Ad26.COV2.S)) OR ("NVX-CoV2373")) OR ("Ad5-based COVID-19 vaccine")) OR ("Gam-COVID-Vac") \| \| **1** \| ((((((("omicron") OR ("B.1.1.529")) OR ("BA.1")) OR ("BA.1.1")) OR ("BA.2")) OR ("BA.2.12.1")) OR ("BA.4")) OR ("BA.5") \| |
| --- | --- | --- | --- | --- | --- | --- | --- | --- | --- | --- | --- | --- | --- | --- | --- | --- | --- | --- | --- | --- | --- | --- | --- |
| **Embase** | 1 ("omicron" or "B.1.1.529" or "BA.1" or "BA.1.1" or "BA.2" or "BA.3" or "BA.4" or "BA.5").mp. [mp=title, abstract, heading word, drug trade name, original title, device manufacturer, drug manufacturer, device trade name, keyword heading word, floating subheading word, candidate term word] (5100)  2 ("mRNA vaccines" or "ChAdOx1-S" or "BNT162b2" or "CoronaVac" or "BBIBP-CorV" or "mRNA-1273" or "Ad26.COV2.S" or "NVX-CoV2373" or "Sputnik V" or "Ad5-based COVID-19 vaccine").mp. [mp=title, abstract, heading word, drug trade name, original title, device manufacturer, drug manufacturer, device trade name, keyword heading word, floating subheading word, candidate term word] (7031)  3 ("PF-07321332" or "Remdesivir" or "EIDD-2801" or "Oseltamivir" or "Favipravir" or " arbidol hydrochloride" or "Ribavirin").mp. [mp=title, abstract, heading word, drug trade name, original title, device manufacturer, drug manufacturer, device trade name, keyword heading word, floating subheading word, candidate term word] (65161)  4 "Azithromycin".mp. (48661)  5 ("dexamethasone" or "hydrocortisone" or "prednisolone" or "prednisone").mp. [mp=title, abstract, heading word, drug trade name, original title, device manufacturer, drug manufacturer, device trade name, keyword heading word, floating subheading word, candidate term word] (651568)  6 ("sarilumab" or "tocilizumab" or "siltuximab" or "Baricitinib" or "Ruxolitinib" or "Tofacitinib" or "Hydroxychloroquine" or "Interferon").mp. [mp=title, abstract, heading word, drug trade name, original title, device manufacturer, drug manufacturer, device trade name, keyword heading word, floating subheading word, candidate term word] (511833)  7 ("Sotrovimab" or "Casirivimab" or "Imdevimab" or "Bamlanivimab" or "AZD7442" or "Ivermectin" or "Fluvoxamine").mp. [mp=title, abstract, heading word, drug trade name, original title, device manufacturer, drug manufacturer, device trade name, keyword heading word, floating subheading word, candidate term word] (31595)  8 2 or 3 or 4 or 5 or 6 or 7 (1226850)  9 ("Mortality" or "Mechanical ventilation" or "Hospital admission" or " Time to symptom resolution" or "Viral clearance" or "Time to clinical improvement" or "Duration of hospitalization" or " Time to viral clearance" or "symptomatic infection" or "ICU Admission" or "clinical recovery" or "progression to severe disease" or "discharge rates" or "COVID-19 infection").mp. [mp=title, abstract, heading word, drug trade name, original title, device manufacturer, drug manufacturer, device trade name, keyword heading word, floating subheading word, candidate term word] (2103567)  10 1 and 8 and 9 (126)  11 limit 10 to yr="2022 -Current" (118)  12 limit 11 to human (114)  13 limit 12 to english language (114)  14 limit 13 to covid-19 (114)  15 "BA.2.12.1".mp. (13)  16 1 or 15 (5100)  17 8 and 9 and 16 (126)  18 limit 17 to (human and english language and yr="2022 -Current" and covid-19) (114) |
| **Cochrane COVID-19 study register** | Filtered by  "omicron" or "B.1.1.529"  Report Results  Treatment And Management |
| **WHO database** | (((omicron) OR (b.1.1.529) OR (ba.1) OR (ba.1.1) OR (ba.2) OR (ba.2.12.1) OR (ba.3) OR (ba.4) OR (ba.5)) AND ( (oral antiviral) OR (pf-07321332) OR (remdesivir) OR (eidd-2801) OR (paxlovid) OR (molnupiravir) OR (nirmatrelvir/ritonavir) OR (sotrovimab) OR (casirivimab) OR (imdevimab) OR (bamlanivimab) OR (azd7442) OR (tixagevimab/cilgavimab) OR (chadox1-s) OR (bnt162b2) OR (mrna-1273) OR (coronavac) OR (bbibp-corv) OR (ad26.cov2.s) OR (nvx-cov2373) OR (ad5-ncov-vaccine) OR (sputnik v) OR (dexamethasone) OR (prednisolone) OR (prednisone) OR (hydrocortisone) OR (sarilumab) OR (tocilizumab) OR (siltuximab) OR (baricitinib) OR (tofacitinib) OR (interferon) OR (ivermectin) OR (fluvoxamine)) ) AND fulltext:("1" OR "1" OR "1" OR "1") AND type_of_study:("rct" OR "observational_studies" OR "clinical_trials") AND covidwho_topics:("variants_concern" OR "vaccines") AND la:("en") AND year_cluster:("2022") AND (year_cluster:[2022 TO 2022]) AND type_of_study:("rct" OR "observational_studies" OR "clinical_trials") AND covidwho_topics:("variants_concern" OR "vaccines") AND la:("en") AND year_cluster:("2022") AND (year_cluster:[2022 TO 2022]) AND (year_cluster:[2022 TO 2022]) AND (year_cluster:[2022 TO 2022]) AND type_of_study:("rct" OR "observational_studies" OR "clinical_trials") AND covidwho_topics:("vaccines" OR "variants_concern") AND la:("en") AND year_cluster:("2022") AND (year_cluster:[2022 TO 2022]) AND (year_cluster:[2022 TO 2022]) AND type_of_study:("rct" OR "observational_studies" OR "clinical_trials") AND covidwho_topics:("variants_concern" OR "vaccines") AND year_cluster:("2022") AND (year_cluster:[2022 TO 2022]) |
