# Supplementary material for: Comparative Effectiveness of Antivirals and Monoclonal Antibodies for Treating COVID‐19 Patients Infected With Omicron Variant: A Systematic Review and Network Meta‐Analysis
Source: Influenza Other Respir Viruses. 2024 Dec 25;18(12):e70065. doi: 10.1111/irv.70065 (PMC11669747; doi:10.1111/irv.70065)
Supplement: Supplementary file 2 — Appendix S2 COVID drug NMA R code. [file IRV-18-e70065-s001.docx]

**Appendix 2**

**COVID drug NMA R code**

#install and load packages

install.packages(c("remotes", "knitr"))

remotes::install_github("audrey-b/BUGSnet@v1.1.0", upgrade = TRUE, build_vignettes = TRUE, dependencies = TRUE)

install.packages("rlang")

install.packages('rjags')

install.packages('gemtc')

install.packages('readxl')

install.packages('stringr')

if (!require("devtools")) {

install.packages("devtools")

}

devtools::install_github("MathiasHarrer/dmetar")

install.packages("ggplot2")

install.packages("dmetar")

library(dmetar)

library(coda)

library(readxl)

library(gemtc)

library(rjags)

library(stringr)

library(ggplot2)

library(robvis)

library(tidyverse)

library(igraph)

#load data

data.ab <- read_excel("39_all_1108_updated.xlsx", sheet = "data.ab", na = "NA")

data.re <- read_excel("39_all_1108_updated.xlsx", sheet = "data.re", na = "NA")

# network set up

# change treatments to include only those in the subgroup

treatments <- read.table(textConnection('

id description

"Molnupiravir" "Molnupiravir"

"Nirmatrelvir_ritonavir" "Nirmatrelvir/ritonavir"

"Remdesivir" "Remdesivir"

"Sotrovimab" "Sotrovimab"

"Bebtelovimab" "Bebtelovimab"

"Control" "Control"

'), header=TRUE)

# apply subset for both data.ab and data.re

# overall

# data.ab$overall==1

# age subgroup

# data.ab$old==1

# other subgroups

# data.ab$overall==1 & data.ab$subgp_1==1

# sensitivity

# data.ab$overall==1 & data.ab$studySize>=100

# data.ab$overall==1 & data.ab$criticalBias==0

network <- mtc.network(data.ab=subset(data.ab, data.ab$overall==1 &

data.ab$outcome=="hospitalization", select = c(study,

treatment, responders, sampleSize)),

data.re=subset(data.re, data.re$overall==1 &

data.re$outcome=="hospitalization", select = c(study,

treatment, diff, std.err)),

treatments=treatments)

# plot(network,vertex.color='blue', vertex.label.dist=5, use.description=TRUE, main = "A) Mortality")

plot(network,vertex.color='blue', vertex.label.dist=5, use.description=TRUE, main = "B) Hospitalization")

summary(network)

set.seed(12345)

model <- mtc.model(network,

type="consistency",

likelihood="binom",

link="cloglog",

linearModel="random",

n.chain=4)

# By default, the model will have 4 chains - generate a seed for each

seeds <- sample.int(4, n = .Machine$integer.max)

# Apply JAGS RNG settings to each chain

model$inits <- mapply(c, model$inits, list(

list(.RNG.name="base::Wichmann-Hill", .RNG.seed=seeds[1]),

list(.RNG.name="base::Marsaglia-Multicarry", .RNG.seed=seeds[2]),

list(.RNG.name="base::Super-Duper", .RNG.seed=seeds[3]),

list(.RNG.name="base::Mersenne-Twister", .RNG.seed=seeds[4])), SIMPLIFY=FALSE)

results <- mtc.run(model,n.adapt=20000, n.iter=50000, thin=1)

summary(results)

summary(relative.effect(results, t1="Control"))

#league table

league<- round(exp(relative.effect.table(results)),digits=3)

print(league)

#sucra values

sucra <- rank.probability(results)

plot(sucra)

sucra <- dmetar::sucra(sucra, lower.is.better = TRUE)

print(sucra)

#forest polot

forest(relative.effect(results, t1="Control"), digits=3)

#global inconsistency

model_ume <-mtc.model(network, type="ume", n.chain=4, likelihood="binom", link="cloglog", linearModel="random")

model_ume_results <- mtc.run(model_ume, n.adapt = 20000, n.iter = 50000, thin = 1)

summary(model_ume_results)

#local inconsistency

set.seed(12345)

resultnodesplit <-mtc.nodesplit(network, n.adapt = 20000, n.iter = 50000, thin = 1, n.chain=4,

likelihood="binom", link="cloglog",linearModel="random")

mtc.nodesplit.summary <- summary(resultnodesplit)

plot(summary(resultnodesplit))

# node-splitting models

mtc.nodesplit.comparisons(network)

set.seed(12345)

model.split <- mtc.model(network,

type="nodesplit",

likelihood="binom",

link="cloglog",

linearModel="random",

n.chain=4,

t1 = "Molnupiravir",

t2 = "Nirmatrelvir_ritonavir")

# By default, the model will have 4 chains - generate a seed for each

seeds <- sample.int(4, n = .Machine$integer.max)

# Apply JAGS RNG settings to each chain

model.split$inits <- mapply(c, model.split$inits, list(

list(.RNG.name="base::Wichmann-Hill", .RNG.seed=seeds[1]),

list(.RNG.name="base::Marsaglia-Multicarry", .RNG.seed=seeds[2]),

list(.RNG.name="base::Super-Duper", .RNG.seed=seeds[3]),

list(.RNG.name="base::Mersenne-Twister", .RNG.seed=seeds[4])), SIMPLIFY=FALSE)

results <- mtc.run(model.split,n.adapt=20000, n.iter=50000, thin=1)

node.split.results <- summary(results)

#heterogeneity

resultanohe <- mtc.anohe(network)

print(summary(resultanohe))
