# Supplementary material for: Comparative Effectiveness of Antivirals and Monoclonal Antibodies for Treating COVID‐19 Patients Infected With Omicron Variant: A Systematic Review and Network Meta‐Analysis
Source: Influenza Other Respir Viruses. 2024 Dec 25;18(12):e70065. doi: 10.1111/irv.70065 (PMC11669747; doi:10.1111/irv.70065)
Supplement: Supplementary file 3 — Figure S1 Risk of bias assessment of observational studies using the ROBINS‐I and randomized controlled trial using the RoB 2. Figure S2 Results for hospitalization from node‐splitting approach. Table S1 Number of events and effect estimates for the outcome of a) mortality and b) hospitalization in eligible studies. Table S2. League tables with network meta‐analytic estimates for the outcome of a) mortality and b) hospitalization in sensitivity analyses. Table S3 League tables with network meta‐analytic estimates for the outcome of a) mortality and b) hospitalization in patient subgroups of 1) B.1.1.529 or BA.1 infection, 2) infection with other Omicron subvariants, 3) organ transplant recipients, and 4) non‐organ transplant recipients. [file IRV-18-e70065-s004.docx]

**Supplementary Figure 1: Risk of bias assessment of observational studies using the ROBINS-I and randomized controlled trial using the RoB 2**

**
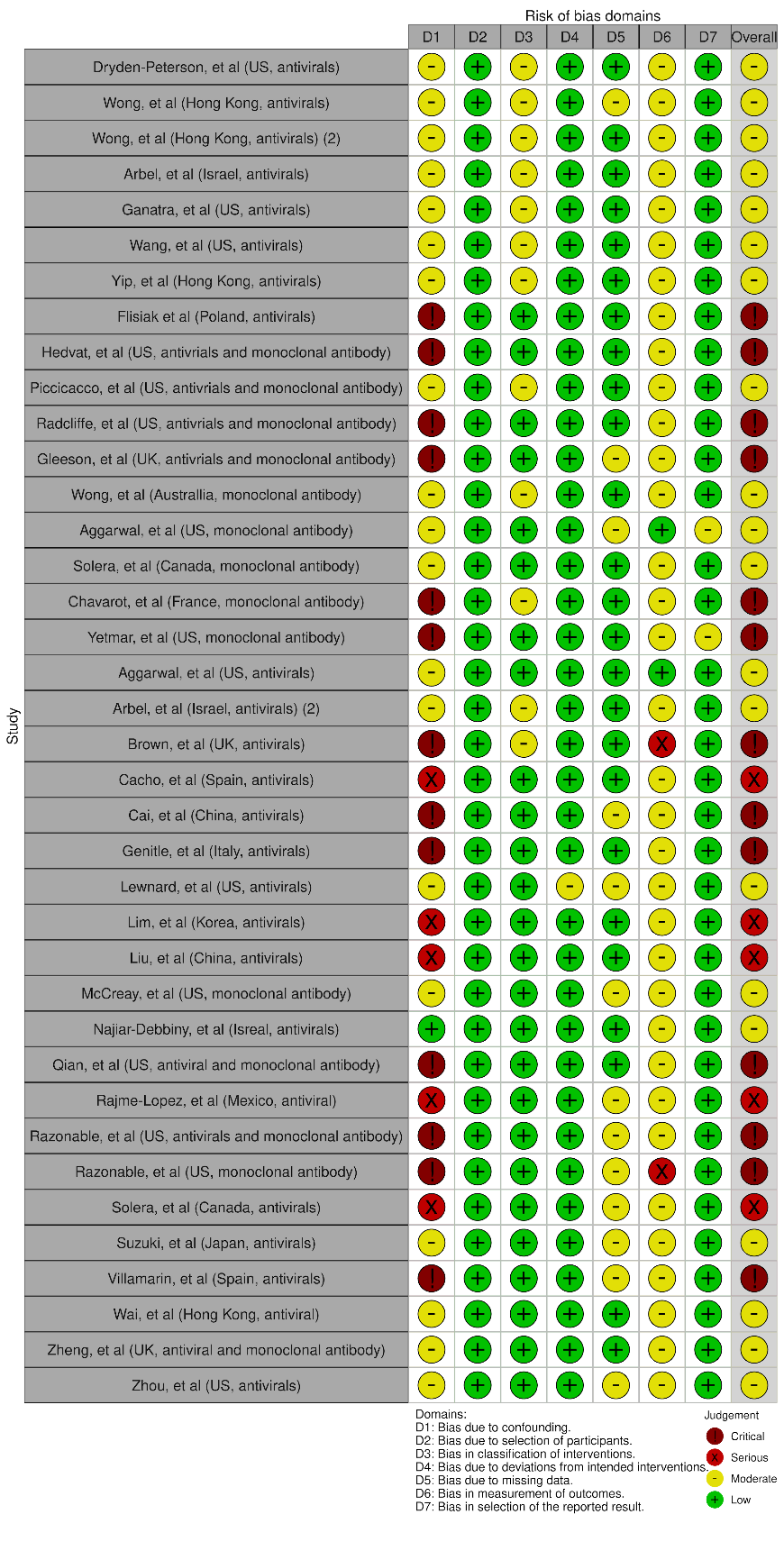
**


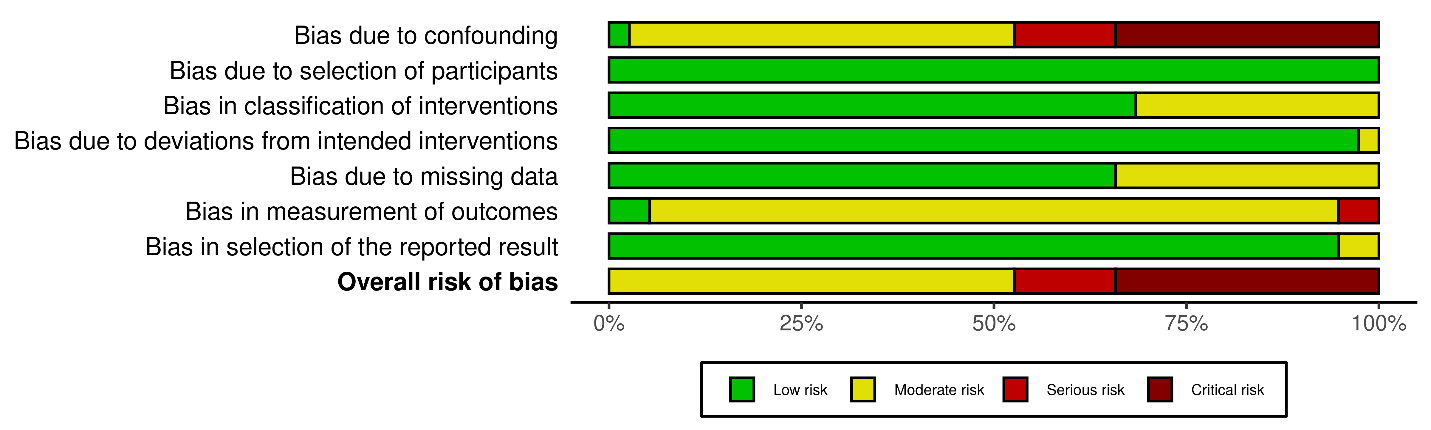


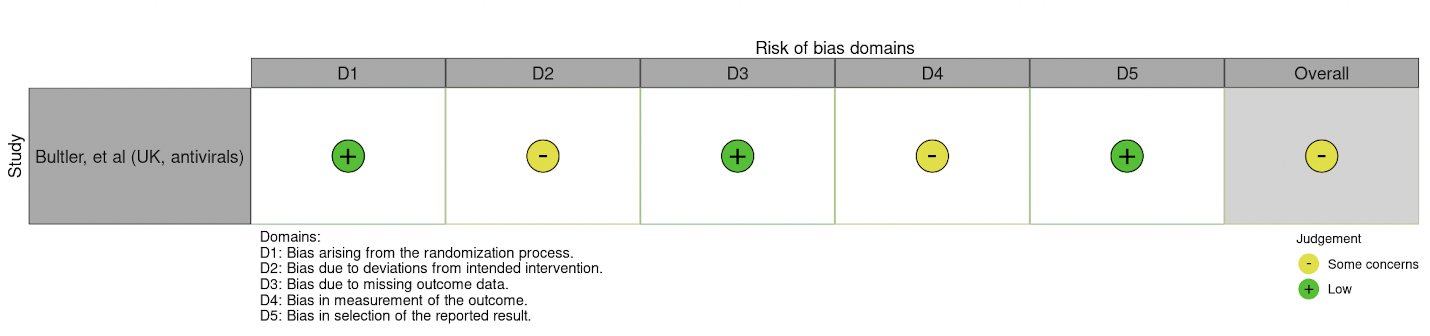


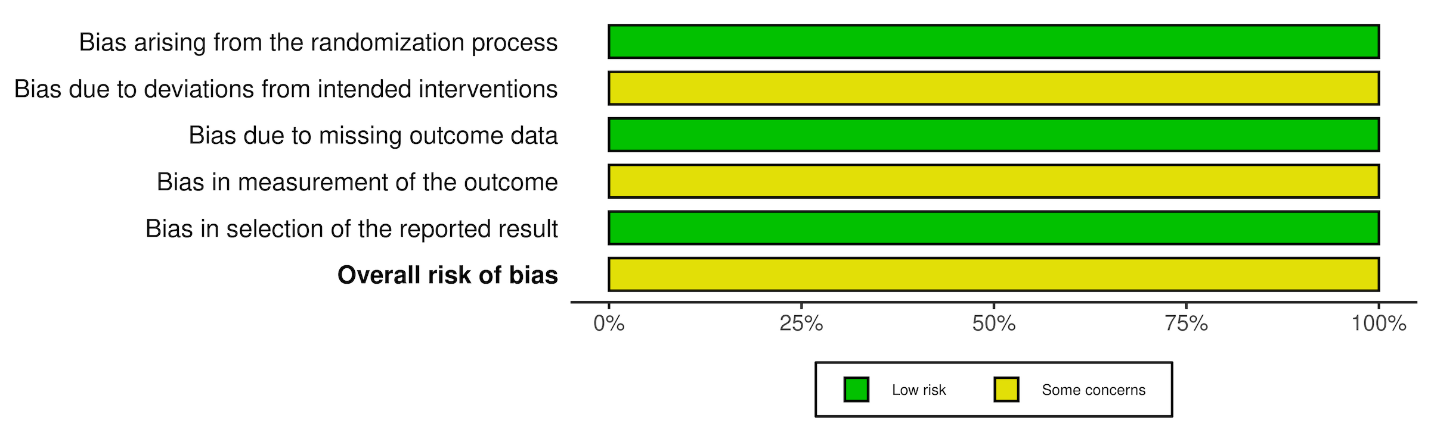


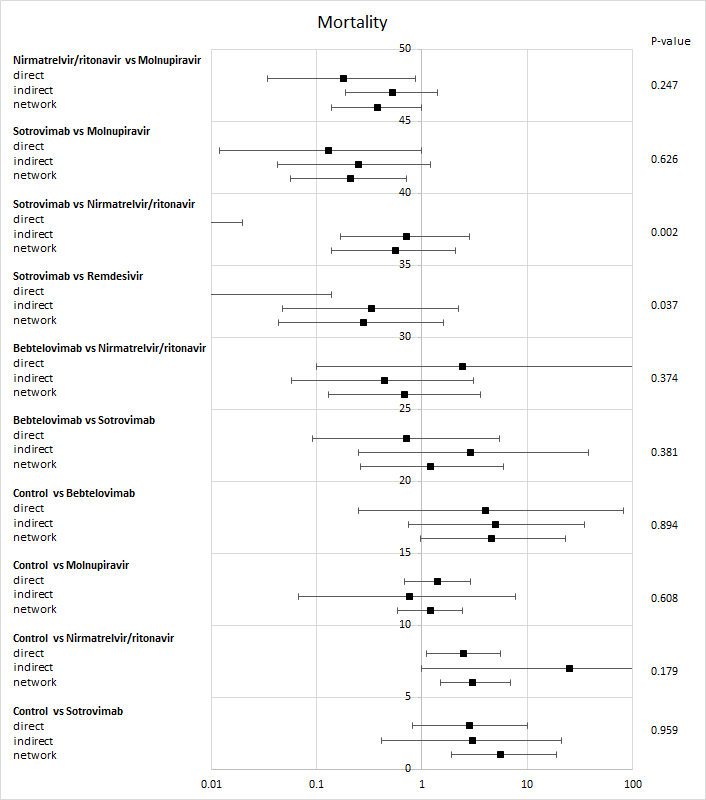
**Supplementary Figure 2:** **Results for hospitalization from node‐splitting approach**


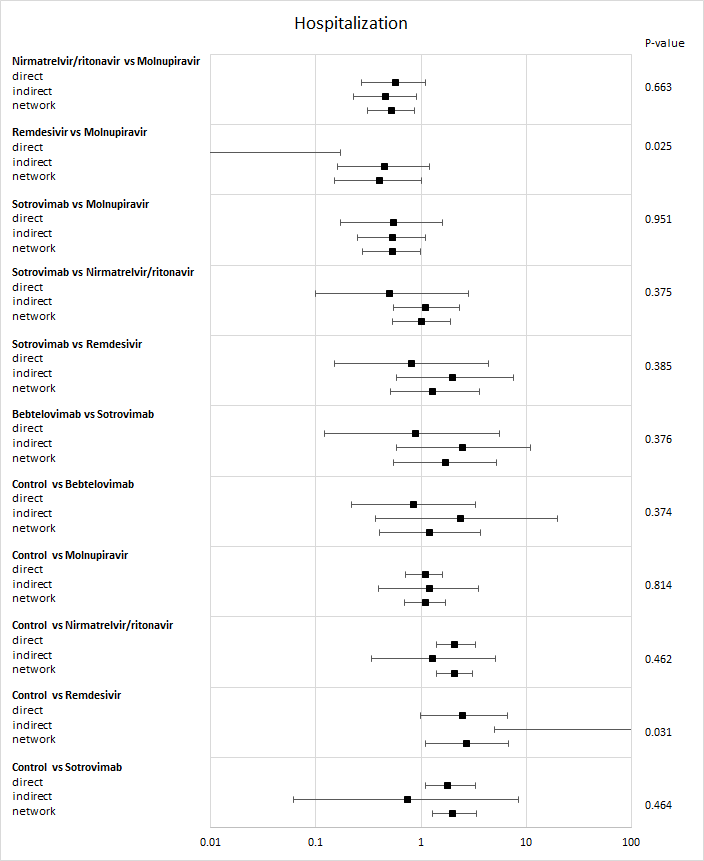


Notes: Comparison of treatments A and B was selected for the node-splitting model if and only if the modified network without studies including both A and B contains a path between A and B.

**Supplementary Table 1. Number of events and effect estimates for the outcome of a) mortality and b) hospitalization in eligible studies**

| A) Mortality | | | | | |
| --- | --- | --- | --- | --- | --- |
| Author's last name (country) | Treatment | Number of  patients | Number of  events | HR | 95% CI |
| Gleeson et al. (UK) | Molnupiravir | 21 | 1 | NA | NA |
|  | Sotrovimab | 47 | 0 | NA | NA |
|  | Control | 48 | 2 | NA | NA |
| Flisiak et al. (Poland) | Molnupiravir | 203 | 20 | NA | NA |
|  | Control | 387 | 63 | NA | NA |
| Radcliffe et al. (USA) | Molnupiravir | 49 | 0 | NA | NA |
|  | Sotrovimab | 24 | 0 | NA | NA |
|  | Nirmatrelvir/ritonavir | 1 | 0 | NA | NA |
|  | Control | 48 | 3 | NA | NA |
| Aggarwal et al. (USA) | Sotrovimab | 1,542 | 1 | NA | NA |
|  | Control | 3,663 | 7 | NA | NA |
| Solera et al. (Canada) | Sotrovimab | 106 | 0 | NA | NA |
|  | Control | 187 | 13 | NA | NA |
| Chavarot et al. (France) | Sotrovimab | 25 | 0 | NA | NA |
|  | Control | 100 | 11 | NA | NA |
| Yetmar et al. (USA) | Sotrovimab | 269 | 2 | NA | NA |
|  | Bebtelovimab | 92 | 2 | NA | NA |
| Ganatra et al. (USA) | Nirmatrelvir/ritonavir | 1,130 | 0 | NA | NA |
|  | Control | 1,130 | 10 | NA | NA |
| Hedvat et al. (USA) | Nirmatrelvir/ritonavir | 28 | 0 | NA | NA |
|  | Sotrovimab | 51 | 0 | NA | NA |
|  | Control | 75 | 3 | NA | NA |
| Piccicacco et al. (USA) | Remdesivir | 82 | 0 | NA | NA |
|  | Sotrovimab | 88 | 0 | NA | NA |
|  | Control | 90 | 1 | NA | NA |
| Butler et al. (UK) | Molnupiravir | 12,516 | 2 | NA | NA |
|  | Control | 12,484 | 5 | NA | NA |
| Lim et al. (South Korea) | Remdesivir | 44 | 1 | NA | NA |
|  | Control | 74 | 5 | NA | NA |
| Razonable et al. (US) | Bebtelovimab | 2,833 | 6 | NA | NA |
|  | Nirmatrelvir/ritonavir | 774 | 0 | NA | NA |
| Liu et al. (China) | Molnupiravir | 26 | 0 | NA | NA |
|  | Control | 16 | 0 | NA | NA |
| Rajme-López et al. (Mexico) | Remdesivir | 54 | 0 | NA | NA |
|  | Control | 72 | 9 | NA | NA |
| Cai et al. (China) | Nirmatrelvir/ritonavir | 61 | 18 | NA | NA |
|  | Control | 43 | 14 | NA | NA |
| McCreary et al. (US) | Bebtelovimab | 1,006 | 1 | NA | NA |
|  | Control | 2,023 | 13 | NA | NA |
| Zheng et al. (UK) | Molnupiravir | 2,689 | 18 | NA | NA |
|  | Sotrovimab | 3,331 | 7 | NA | NA |
| Wai et al. (HK, China) | Molnupiravir | 6,144 | 76 | NA | NA |
|  | Nirmatrelvir/ritonavir | 4,724 | 12 | NA | NA |
|  | Control | 43,487 | 5,276 | NA | NA |
| Gentile et al. (Italy) | Molnupiravir | 146 | 1 | NA | NA |
|  | Nirmatrelvir/ritonavir | 111 | 0 | NA | NA |
| Qian et al. (US) | Molnupiravir | 105 | 0 | NA | NA |
|  | Nirmatrelvir/ritonavir | 307 | 1 | NA | NA |
|  | Control | 278 | 2 | NA | NA |
| Razonable et al. (US) | Bebtelovimab | 1,690 | 3 | NA | NA |
|  | Sotrovimab | 2,182 | 9 | NA | NA |
| Suzuki et al. (Japan) | Molnupiravir | 281 | 3 | NA | NA |
|  | Control | 1,636 | 21 | NA | NA |
| Zhou et al. (US) | Nirmatrelvir/ritonavir | 2,811 | 7 | NA | NA |
|  | Control | 194,542 | 100 | NA | NA |
| Aggarwal et al. (USA) | Nirmatrelvir/ritonavir | 3,614 | 0 | NA | NA |
|  | Control | 4,835 | 9 | NA | NA |
| Solera et al. (Canada) | Remdesivir | 86 | 0 | NA | NA |
|  | Control | 106 | 2 | NA | NA |
| Cacho et al. (Spain) | Remdesivir | 57 | 5 | NA | NA |
|  | Control | 41 | 2 | NA | NA |
| Wong et al. (HK, China) | Molnupiravir | 4,983 | 88 | 0.76 | (0.61, 0.95) |
|  | Control | 49,234 | 1,136 | (reference) | |
| Wong et al. (HK, China) | Nirmatrelvir/ritonavir | 5,542 | 22 | 0.34 | (0.22, 0.52) |
|  | Control | 54,672 | 637 | (reference) | |
| Wong et al. (HK, China) | Molnupiravir | 1,856 | 150 | 0.48 | (0.40, 0.59) |
|  | Control | 1,856 | 295 | (reference) | |
| Wong et al. (HK, China) | Nirmatrelvir/ritonavir | 890 | 32 | 0.34 | (0.23, 0.50) |
|  | Control | 890 | 92 | (reference) | |
| Arbel et al. (Israel)  (Age 40-64) | Nirmatrelvir/ritonavir | 1,418 | 1 | 1.32 | (0.16, 10.75) |
|  | Control | 65,015 | 16 | (reference) | |
| Arbel et al. (Israel)  (Age≥65) | Nirmatrelvir/ritonavir | 2,484 | 2 | 0.21 | (0.05, 0.82) |
|  | Control | 40,337 | 158 | (reference) | |
| Arbel et al. (Israel)  (Age 40-64) | Molnupiravir | 224 | 4 | 12.82 | (3.41, 48.17) |
|  | Control | 6,075 | 7 | (reference) | |
| Arbel et al. (Israel)  (Age≥65) | Molnupiravir | 845 | 5 | 0.26 | (0.10, 0.73) |
|  | Control | 12,724 | 137 | (reference) | |
| Najjar-Debbiny et al. (Israel) | Molnupiravir | 2,661 | 22 | 0.81 | (0.46, 1.43) |
|  | Control | 2,661 | 27 | (reference) | |
|  |  |  |  |  |  |
| B) Hospitalization | | | | | |
| Author's last name (country) | Treatment | Number of  patients | Number of  events | HR | 95% CI |
| Dryden-Peterson et al. (USA) | Nirmatrelvir/ritonavir | 6,036 | 40 | NA | NA |
|  | Control | 24,286 | 232 | NA | NA |
| Gleeson et al. (UK) | Molnupiravir | 21 | 3 | NA | NA |
|  | Sotrovimab | 47 | 1 | NA | NA |
|  | Control | 48 | 10 | NA | NA |
| Radcliffe et al. (USA) | Molnupiravir | 49 | 8 | NA | NA |
|  | Sotrovimab | 24 | 2 | NA | NA |
|  | Nirmatrelvir/ritonavir | 1 | 0 | NA | NA |
|  | Control | 48 | 13 | NA | NA |
| Wong et al. (Australia) | Sotrovimab | 27 | 10 | NA | NA |
|  | Control | 14 | 13 | NA | NA |
| Aggarwal et al. (USA) | Sotrovimab | 1,542 | 39 | NA | NA |
|  | Control | 3,663 | 116 | NA | NA |
| Solera et al. (Canada) | Sotrovimab | 106 | 17 | NA | NA |
|  | Control | 187 | 52 | NA | NA |
| Chavarot et al. (France) | Sotrovimab | 25 | 4 | NA | NA |
|  | Control | 100 | 35 | NA | NA |
| Yetmar et al. (USA) | Sotrovimab | 269 | 8 | NA | NA |
|  | Bebtelovimab | 92 | 3 | NA | NA |
| Ganatra et al. (USA) | Nirmatrelvir/ritonavir | 1,130 | 10 | NA | NA |
|  | Control | 1,130 | 23 | NA | NA |
| Hedvat et al. (USA) | Nirmatrelvir/ritonavir | 28 | 3 | NA | NA |
|  | Sotrovimab | 51 | 5 | NA | NA |
|  | Control | 75 | 23 | NA | NA |
| Piccicacco et al. (USA) | Remdesivir | 82 | 7 | NA | NA |
|  | Sotrovimab | 88 | 7 | NA | NA |
|  | Control | 90 | 11 | NA | NA |
| Butler et al. (UK) | Molnupiravir | 12,516 | 102 | NA | NA |
|  | Control | 12,484 | 93 | NA | NA |
| Brown et al. (UK) | Molnupiravir | 442 | 8 | NA | NA |
|  | Sotrovimab | 186 | 6 | NA | NA |
|  | Control | 4,160 | 35 | NA | NA |
| Rajme-López et al. (Mexico) | Remdesivir | 54 | 5 | NA | NA |
|  | Control | 72 | 22 | NA | NA |
| McCreary et al. (US) | Bebtelovimab | 1,006 | 33 | NA | NA |
|  | Control | 2,023 | 58 | NA | NA |
| Lewnard et al. (US) | Nirmatrelvir/ritonavir | 4,329 | 5 | NA | NA |
|  | Control | 20,980 | 27 | NA | NA |
| Gentile et al. (Italy) | Molnupiravir | 146 | 3 | NA | NA |
|  | Nirmatrelvir/ritonavir | 111 | 1 | NA | NA |
| Qian et al. (US) | Molnupiravir | 105 | 5 | NA | NA |
|  | Nirmatrelvir/ritonavir | 307 | 4 | NA | NA |
|  | Control | 278 | 49 | NA | NA |
| Villamarín et al. (Spain) | Molnupiravir | 9 | 1 | NA | NA |
|  | Remdesivir | 7 | 0 | NA | NA |
| Aggarwal et al. (USA) | Nirmatrelvir/ritonavir | 3,614 | 31 | NA | NA |
|  | Control | 4,835 | 64 | NA | NA |
| Solera et al. (Canada) | Remdesivir | 86 | 2 | NA | NA |
|  | Control | 106 | 13 | NA | NA |
| Wong et al. (HK, China) | Molnupiravir | 4,983 | 485 | 0.98 | (0.89, 1.06) |
|  | Control | 49,234 | 4,931 | (reference) | |
| Wong et al. (HK, China) | Nirmatrelvir/ritonavir | 5,542 | 246 | 0.76 | (0.67, 0.86) |
|  | Control | 54,672 | 3,186 | (reference) | |
| Arbel et al. (Israel)  (Age 40-64) | Nirmatrelvir/ritonavir | 1,418 | 7 | 0.74 | (0.35, 1.58) |
|  | Control | 65,015 | 327 | (reference) | |
| Arbel et al. (Israel)  (Age≥65) | Nirmatrelvir/ritonavir | 2,484 | 11 | 0.27 | (0.15, 0.49) |
|  | Control | 40,337 | 766 | (reference) | |
| Wang et al. (USA) | Nirmatrelvir/ritonavir | 2,226 | 27 | 0.92 | (0.56, 1.55) |
|  | Molnupiravir | 2,226 | 31 | (reference) | |
| Yip et al. (HK, China) | Molnupiravir | 4,921 | 437 | 1.17 | (0.99, 1.39) |
|  | Nirmatrelvir/ritonavir | 5,808 | 172 | 0.79 | (065, 0.95) |
|  | Control | 83,154 | 1,322 | (reference) | |
| Arbel et al. (Israel)  (Age 40-64) | Molnupiravir | 224 | 8 | 1.80 | (0.86, 3.77) |
|  | Control | 6,075 | 97 | (reference) | |
| Arbel et al. (Israel)  (Age≥65) | Molnupiravir | 845 | 18 | 0.55 | (0.34, 0.88) |
|  | Control | 12,724 | 513 | (reference) | |
| Zhou et al. (US) | Nirmatrelvir/ritonavir | 2,808 | 34 | 0.16 | (0.11, 0.22) |
|  | Control | 10,849 | 752 | (reference) | |

Note: HR=Hazard ratio; CI=Confidence interval

**Supplementary Table 2. League tables with network meta-analytic estimates for the outcome of a) mortality and b) hospitalization in sensitivity analyses**

| **Excluding small studies with sample size less than 100** | | | | | |
| --- | --- | --- | --- | --- | --- |
| A) Mortality | | | | | |
| Molnupiravir | 0.395  (0.143, 1.034) | 0.512  (0.083, 2.908) | **0.22**  **(0.058, 0.721)** | 0.27  (0.049, 1.399) | 1.248  (0.607, 2.516) |
| 2.532  (0.967, 6.985) | Nirmatrelvir/  ritonavir | 1.3  (0.207, 7.796) | 0.558  (0.14, 2.034) | 0.682  (0.13, 3.528) | **3.164**  **(1.477, 6.957)** |
| 1.953  (0.344, 11.98) | 0.769  (0.128, 4.84) | Remdesivir | 0.429  (0.058, 3.021) | 0.528  (0.055, 5.075) | 2.43  (0.493, 13.04) |
| **4.547**  **(1.388, 17.21)** | 1.794  (0.492, 7.166) | 2.331  (0.331, 17.18) | Sotrovimab | 1.233  (0.267, 5.862) | **5.659**  **(1.894, 19.33)** |
| 3.708  (0.715, 20.51) | 1.466  (0.283, 7.692) | 1.895  (0.197, 18.1) | 0.811  (0.171, 3.749) | Bebtelovimab | 4.631  (0.98, 22.86) |
| 0.801  (0.398, 1.648) | **0.316**  **(0.144, 0.677)** | 0.412  (0.077, 2.029) | **0.177**  **(0.052, 0.528)** | 0.216  (0.044, 1.021) | Control |
|  |  |  |  |  |  |
|  |  |  |  |  |  |
| B) Hospitalization | | | | | |
| Molnupiravir | **0.527**  **(0.313, 0.875)** | 0.448  (0.164, 1.198) | 0.6  (0.314, 1.119) | 0.969  (0.294, 2.993) | 1.098  (0.71, 1.685) |
| **1.896**  **(1.143, 3.198)** | Nirmatrelvir/  ritonavir | 0.85  (0.318, 2.259) | 1.139  (0.605, 2.128) | 1.836  (0.573, 5.667) | **2.08**  **(1.412, 3.108)** |
| 2.232  (0.835, 6.088) | 1.177  (0.443, 3.144) | Remdesivir | 1.341  (0.498, 3.605) | 2.159  (0.53, 8.53) | **2.45**  **(1.007, 6.065)** |
| 1.665  (0.894, 3.189) | 0.878  (0.47, 1.653) | 0.746  (0.277, 2.008) | Sotrovimab | 1.614  (0.519, 4.846) | **1.828**  **(1.1, 3.093)** |
| 1.032  (0.334, 3.398) | 0.545  (0.176, 1.745) | 0.463  (0.117, 1.886) | 0.62  (0.206, 1.926) | Bebtelovimab | 1.133  (0.395, 3.42) |
| 0.911  (0.593, 1.408) | **0.481**  **(0.322, 0.708)** | **0.408**  **(0.165, 0.993)** | **0.547**  **(0.323, 0.909)** | 0.883  (0.292, 2.531) | Control |
|  |  |  |  |  |  |

| **Excluding studies with critical risk of bias** | | | | | |
| --- | --- | --- | --- | --- | --- |
| A) Mortality | | | | | |
| Molnupiravir | 0.364  (0.09, 1.409) | 0.801  (0.126, 4.917) | 0.245  (0.035, 1.459) | 0.309  (0.01, 8.289) | 1.243  (0.466, 3.204) |
| 2.745  (0.71, 11.1) | Nirmatrelvir/  ritonavir | 2.213  (0.328, 14.54) | 0.678  (0.079, 4.97) | 0.853  (0.026, 23.87) | **3.412**  **(1.18, 9.878)** |
| 1.248  (0.203, 7.961) | 0.452  (0.069, 3.048) | Remdesivir | 0.306  (0.028, 2.946) | 0.385  (0.01, 13.26) | 1.547  (0.326, 7.525) |
| 4.074  (0.686, 28.59) | 1.475  (0.201, 12.7) | 3.267  (0.339, 35.65) | Sotrovimab | 1.264  (0.031, 50.41) | 5.026  (0.908, 32.93) |
| 3.233  (0.121, 103.6) | 1.172  (0.042, 38.38) | 2.598  (0.075, 101.2) | 0.791  (0.02, 32.7) | Bebtelovimab | 4.01  (0.171, 110.9) |
| 0.804  (0.312, 2.147) | **0.293**  **(0.101, 0.847)** | 0.646  (0.133, 3.067) | 0.199  (0.03, 1.102) | 0.249  (0.009, 5.86) | Control |
|  |  |  |  |  |  |
|  |  |  |  |  |  |
| B) Hospitalization | | | | | |
| Molnupiravir | 0.606  (0.355, 1.03) | 0.437  (0.168, 1.105) | 0.53  (0.238, 1.132) | 1.274  (0.367, 4.398) | 1.087  (0.682, 1.725) |
| 1.65  (0.971, 2.814) | Nirmatrelvir/  ritonavir | 0.721  (0.288, 1.751) | 0.876  (0.411, 1.786) | 2.106  (0.622, 7.033) | **1.794**  **(1.231, 2.61)** |
| 2.287  (0.905, 5.967) | 1.386  (0.571, 3.47) | Remdesivir | 1.212  (0.463, 3.153) | 2.923  (0.717, 12.03) | **2.484**  **(1.114, 5.734)** |
| 1.885  (0.883, 4.209) | 1.142  (0.56, 2.436) | 0.825  (0.317, 2.159) | Sotrovimab | 2.404  (0.661, 9.057) | **2.049**  **(1.117, 3.913)** |
| 0.785  (0.227, 2.723) | 0.475  (0.142, 1.609) | 0.342  (0.083, 1.395) | 0.416  (0.11, 1.512) | Bebtelovimab | 0.852  (0.272, 2.704) |
| 0.92  (0.58, 1.467) | **0.557**  **(0.383, 0.812)** | **0.403**  **(0.174, 0.898)** | **0.488**  **(0.256, 0.896)** | 1.174  (0.37, 3.683) | Control |
|  |  |  |  |  |  |

Note: NA=not applicable when the hazard ratios were not estimable. When excluding small studies with sample size less than 100, 30 and 25 studies were included in the analyses for the outcome of (A) mortality and (B) hospitalization, respectively. When excluding studies with critical risk of bias, 21 and 18 studies were included in the analyses for the outcome of (A) mortality and (B) hospitalization, respectively. Comparisons should be read from top to bottom and from left to right. Results are the hazard ratios with 95% credible interval in the column-defining therapy compared with the row-defining therapy. Values in bold are ones where the 95% credible intervals do not include 1 (null effect).

**Supplementary Table 3. League tables with network meta-analytic estimates for the outcome of a) mortality and b) hospitalization in patient subgroups of 1) B.1.1.529 or BA.1 infection, 2) infection with other Omicron subvariants, 3) organ transplant recipients, and 4) non-organ transplant recipients**

| 1. **B.1.1.529 or BA.1 infection** | | | | | |
| --- | --- | --- | --- | --- | --- |
| A) Mortality | | | | | |
| Molnupiravir | 0.343  (0.043, 2.676) | 1.515  (0.016, 161.6) | **0.153**  **(0.021, 0.664)** | 0.276  (0.005, 9.243) | 1.054  (0.289, 3.692) |
| 2.913  (0.374, 23.19) | Nirmatrelvir/  ritonavir | 4.399  (0.04, 524.1) | 0.444  (0.038, 3.236) | 0.807  (0.011, 34.69) | 3.063  (0.583, 15.82) |
| 0.66  (0.006, 63.67) | 0.227  (0.002, 25.06) | Remdesivir | 0.099  (0.001, 8.34) | 0.18  (0, 43.52) | 0.695  (0.008, 56.88) |
| **6.534**  **(1.505, 48.03)** | 2.252  (0.309, 26.07) | 10.14  (0.12, 1282) | Sotrovimab | 1.832  (0.06, 50.34) | **6.86**  **(1.929, 40.59)** |
| 3.623  (0.108, 204.6) | 1.239  (0.029, 90.85) | 5.542  (0.023, 2151) | 0.546  (0.02, 16.7) | Bebtelovimab | 3.828  (0.12, 193.8) |
| 0.949  (0.271, 3.463) | 0.326  (0.063, 1.715) | 1.438  (0.018, 130.6) | **0.146**  **(0.025, 0.518)** | 0.261  (0.005, 8.302) | Control |
|  |  |  |  |  |  |
|  |  |  |  |  |  |
| B) Hospitalization | | | | | |
| Molnupiravir | 0.442  (0.156, 1.248) | 0.671  (0.127, 3.393) | 0.486  (0.21, 1.082) | 0.429  (0.046, 3.318) | 1.001  (0.5, 2.021) |
| 2.263  (0.801, 6.396) | Nirmatrelvir/  ritonavir | 1.521  (0.276, 8.107) | 1.099  (0.427, 2.73) | 0.97  (0.101, 7.844) | **2.268**  **(1.044, 4.969)** |
| 1.489  (0.295, 7.852) | 0.657  (0.123, 3.621) | Remdesivir | 0.723  (0.16, 3.352) | 0.639  (0.05, 7.202) | 1.492  (0.338, 6.901) |
| 2.059  (0.924, 4.763) | 0.91  (0.366, 2.34) | 1.383  (0.298, 6.25) | Sotrovimab | 0.888  (0.112, 5.822) | **2.06**  **(1.211, 3.679)** |
| 2.333  (0.301, 21.77) | 1.03  (0.127, 9.898) | 1.565  (0.139, 20.1) | 1.126  (0.172, 8.902) | Bebtelovimab | 2.324  (0.334, 19.98) |
| 0.999  (0.495, 2) | **0.441**  **(0.201, 0.958)** | 0.67  (0.145, 2.958) | **0.485**  **(0.272, 0.826)** | 0.43  (0.05, 2.998) | Control |
|  |  |  |  |  |  |
|  |  |  |  |  |  |
| 1. **Infection with other Omicron subvariants** | | | | | |
| A) Mortality | | | | | |
| Molnupiravir | 0.274  (0.023, 2.223) | 0.882  (0.005, 77.44) | NA | 0.688  (0.006, 107.3) | 1.866  (0.246, 9.592) |
| 3.654  (0.45, 43.65) | Nirmatrelvir/  ritonavir | 3.259  (0.021, 319.9) | NA | 2.51  (0.048, 269.2) | **6.811**  **(1.218, 38.46)** |
| 1.134  (0.013, 211.5) | 0.307  (0.003, 47.41) | Remdesivir | NA | 0.834  (0.002, 702.3) | 2.076  (0.03, 239.1) |
| NA | NA | NA | Sotrovimab | NA | NA |
| 1.454  (0.009, 165.5) | 0.398  (0.004, 20.99) | 1.199  (0.001, 552) | NA | Bebtelovimab | 2.703  (0.019, 206.3) |
| 0.536  (0.104, 4.064) | **0.147**  **(0.026, 0.821)** | 0.482  (0.004, 33.21) | NA | 0.37  (0.005, 52.98) | Control |
|  |  |  |  |  |  |
|  |  |  |  |  |  |
| B) Hospitalization | | | | | |
| Molnupiravir | 0.701  (0.5, 1.001) | **0.233**  **(0.045, 0.825)** | NA | NA | 0.943  (0.691, 1.263) |
| 1.427  (0.999, 2.002) | Nirmatrelvir/  ritonavir | 0.332  (0.064, 1.162) | NA | NA | **1.345**  **(1.022, 1.71)** |
| **4.292**  **(1.213, 22.35)** | 3.011  (0.861, 15.61) | Remdesivir | NA | NA | **4.029**  **(1.178, 20.29)** |
| NA | NA | NA | Sotrovimab | NA | NA |
| NA | NA | NA | NA | Bebtelovimab | NA |
| 1.06  (0.792, 1.448) | **0.744**  **(0.585, 0.978)** | **0.248**  **(0.049, 0.849)** | NA | NA | Control |
|  |  |  |  |  |  |
|  |  |  |  |  |  |
| 1. **Organ transplant recipients** | | | | | |
| A) Mortality | | | | | |
| Molnupiravir | 0.694  (0.006, 24.96) | 1.49  (0.04, 36.23) | 0.08  (0.002, 1.046) | 0.143  (0.001, 7.259) | 1.067  (0.085, 10.56) |
| 1.44  (0.04, 165.3) | Nirmatrelvir/  ritonavir | 2.163  (0.044, 220.8) | 0.114  (0.002, 6.384) | 0.213  (0.001, 34.42) | 1.494  (0.077, 91.84) |
| 0.671  (0.028, 25.04) | 0.462  (0.005, 22.92) | Remdesivir | **0.054**  **(0.001, 0.85)** | 0.097  (0.001, 6.192) | 0.722  (0.072, 8.458) |
| 12.5  (0.956, 543.5) | 8.807  (0.157, 468.3) | **18.52**  **(1.176, 782.9)** | Sotrovimab | 1.836  (0.062, 49.1) | **13.33**  **(2.426, 223)** |
| 6.992  (0.138, 1302) | 4.7  (0.029, 993.2) | 10.32  (0.161, 1719) | 0.545  (0.02, 16.25) | Bebtelovimab | 7.44  (0.237, 705) |
| 0.938  (0.095, 11.78) | 0.669  (0.011, 13.02) | 1.385  (0.118, 13.89) | **0.075**  **(0.004, 0.412)** | 0.134  (0.001, 4.223) | Control |
|  |  |  |  |  |  |
|  |  |  |  |  |  |
| B) Hospitalization | | | | | |
| Molnupiravir | 0.438  (0.061, 2.058) | 0.23  (0.027, 1.254) | 0.381  (0.104, 1.042) | 0.335  (0.032, 2.387) | 1.214  (0.401, 3.134) |
| 2.283  (0.486, 16.36) | Nirmatrelvir/  ritonavir | 0.531  (0.054, 4.878) | 0.87  (0.207, 4.305) | 0.786  (0.071, 8.187) | 2.759  (0.73, 14.19) |
| 4.342  (0.797, 37.57) | 1.884  (0.205, 18.36) | Remdesivir | 1.634  (0.304, 11.14) | 1.467  (0.118, 18.64) | **5.223**  **(1.144, 34)** |
| 2.626  (0.96, 9.572) | 1.149  (0.232, 4.834) | 0.612  (0.09, 3.294) | Sotrovimab | 0.891  (0.133, 4.986) | **3.161**  **(1.815, 6.573)** |
| 2.987  (0.419, 31.07) | 1.273  (0.122, 14.06) | 0.682  (0.054, 8.498) | 1.122  (0.201, 7.499) | Bebtelovimab | 3.59  (0.601, 28.17) |
| 0.824  (0.319, 2.495) | 0.362  (0.07, 1.37) | **0.191**  **(0.029, 0.874)** | **0.316**  **(0.152, 0.551)** | 0.279  (0.036, 1.664) | Control |
|  |  |  |  |  |  |
|  |  |  |  |  |  |
| 1. **Non-organ transplant recipients** | | | | | |
| A) Mortality | | | | | |
| Molnupiravir | **0.344**  **(0.11, 0.994)** | 0.467  (0.055, 3.648) | 0.41  (0.069, 2.195) | 0.29  (0.039, 2.037) | 1.149  (0.501, 2.518) |
| **2.906**  **(1.006, 9.123)** | Nirmatrelvir/  ritonavir | 1.361  (0.159, 11.3) | 1.194  (0.186, 7.281) | 0.839  (0.123, 5.912) | **3.34**  **(1.447, 8.033)** |
| 2.14  (0.274, 18.27) | 0.735  (0.089, 6.28) | Remdesivir | 0.883  (0.072, 10.58) | 0.623  (0.043, 9.127) | 2.455  (0.36, 17.93) |
| 2.441  (0.456, 14.48) | 0.837  (0.137, 5.368) | 1.133  (0.095, 13.93) | Sotrovimab | 0.709  (0.092, 5.503) | 2.796  (0.54, 15.64) |
| 3.452  (0.491, 25.71) | 1.192  (0.169, 8.113) | 1.604  (0.11, 23.2) | 1.41  (0.182, 10.84) | Bebtelovimab | 3.979  (0.615, 26.26) |
| 0.87  (0.397, 1.997) | **0.299**  **(0.124, 0.691)** | 0.407  (0.056, 2.777) | 0.358  (0.064, 1.853) | 0.251  (0.038, 1.627) | Control |
|  |  |  |  |  |  |
|  |  |  |  |  |  |
| B) Hospitalization | | | | | |
| Molnupiravir | **0.522**  **(0.285, 0.933)** | 0.614  (0.178, 2.092) | 1.085  (0.405, 2.897) | 1.282  (0.271, 5.933) | 1.089  (0.65, 1.807) |
| **1.914**  **(1.072, 3.508)** | Nirmatrelvir/  ritonavir | 1.178  (0.351, 3.976) | 2.076  (0.783, 5.624) | 2.454  (0.533, 11.3) | **2.084**  **(1.343, 3.29)** |
| 1.628  (0.478, 5.62) | 0.849  (0.252, 2.852) | Remdesivir | 1.772  (0.491, 6.407) | 2.088  (0.33, 13.13) | 1.771  (0.577, 5.507) |
| 0.922  (0.345, 2.47) | 0.482  (0.178, 1.277) | 0.564  (0.156, 2.035) | Sotrovimab | 1.184  (0.214, 6.469) | 1.005  (0.415, 2.436) |
| 0.78  (0.169, 3.688) | 0.408  (0.088, 1.876) | 0.479  (0.076, 3.027) | 0.845  (0.155, 4.672) | Bebtelovimab | 0.85  (0.198, 3.679) |
| 0.918  (0.553, 1.538) | **0.48**  **(0.304, 0.745)** | 0.565  (0.182, 1.733) | 0.995  (0.411, 2.407) | 1.177  (0.272, 5.044) | Control |
|  |  |  |  |  |  |

Note: NA=not applicable when the hazard ratios were not estimable. Among subgroups of 1) B.1.1.529 or BA.1 infection, 13 studies were included in each analysis for the outcome of (A) mortality and (B) hospitalization. Among subgroups of 2) infection with other Omicron subvariants, 7 and 5 studies were included in the analyses for the outcome of (A) mortality and (B) hospitalization, respectively. Among subgroups of 3) organ transplant recipients, 8 and 9 studies were included in the analyses for the outcome of (A) mortality and (B) hospitalization, respectively. Among subgroups of 4) non-organ transplant recipients, 24 and 18 studies were included in the analyses for the outcome of (A) mortality and (B) hospitalization, respectively. Comparisons should be read from top to bottom and from left to right. Results are the hazard ratios with 95% credible interval in the column-defining therapy compared with the row-defining therapy. Values in bold are ones where the 95% credible intervals do not include 1 (null effect). Other Omicron subvariants refers to BA.2, BA.4, or BA.5 sublineages.
